# Effects of selenoprotein extracts from *Cardamine hupingshanensis* on growth, selenium metabolism, antioxidant capacity, immunity and intestinal health in largemouth bass *Micropterus salmoides*

**DOI:** 10.3389/fimmu.2024.1342210

**Published:** 2024-01-22

**Authors:** Hao Zhang, Long Zhao, Penghui Zhang, Yuanyuan Xie, Xinfeng Yao, Xuewen Pan, Yifan Fu, Jiao Wei, Hongfeng Bai, Xianping Shao, Jinyun Ye, Chenglong Wu

**Affiliations:** ^1^ National-Local Joint Engineering Laboratory of Aquatic Animal Genetic Breeding and Nutrition (Zhejiang), School of Life Science, Huzhou University, Huzhou, China; ^2^ Zhejiang Provincial Key Laboratory of Aquatic Resources Conservation and Development, School of Life Science, Huzhou University, Huzhou, China

**Keywords:** *Micropterus salmoides*, selenoprotein extracts from *Cardamine hupingshanensis*, hematology, antioxidant capacities, immune and inflammatory responses

## Abstract

This study aimed to assess the impact of dietary selenoprotein extracts from *Cardamine hupingshanensis* (SePCH) on the growth, hematological parameters, selenium metabolism, immune responses, antioxidant capacities, inflammatory reactions and intestinal barrier functions in juvenile largemouth bass (*Micropterus salmoides*). The base diet was supplemented with four different concentrations of SePCH: 0.00, 0.30, 0.60 and 1.20 g/Kg (actual selenium contents: 0.37, 0.59, 0.84 and 1.30 mg/kg). These concentrations were used to formulate four isonitrogenous and isoenergetic diets for juvenile largemouth bass during a 60-day culture period. Adequate dietary SePCH (0.60 and 1.20 g/Kg) significantly increased weight gain and daily growth rate compared to the control groups (0.00 g/Kg). Furthermore, 0.60 and 1.20 g/Kg SePCH significantly enhanced amounts of white blood cells, red blood cells, platelets, lymphocytes and monocytes, and levels of hemoglobin, mean corpuscular volume and mean corpuscular hemoglobin in the hemocytes. In addition, 0.60 and 1.20 g/Kg SePCH increased the mRNA expression levels of selenocysteine lyase, selenophosphate synthase 1, 15 kDa selenoprotein, selenoprotein T2, selenoprotein H, selenoprotein P and selenoprotein K in the fish liver and intestine compared to the controls. Adequate SePCH not only significantly elevated the activities of antioxidant enzymes (Total superoxide dismutase, catalase, glutathione reductase, glutathione peroxidase), the levels of total antioxidant capacity and glutathione, while increased mRNA transcription levels of NF-E2-related factor 2, Cu/Zn-superoxide dismutase, catalase, glutathione reductase and glutathione peroxidase. However, adequate SePCH significantly decreased levels of malondialdehyde and H_2_O_2_ and the mRNA expression levels of kelch-like ECH-associated protein 1a and kelch-like ECH-associated protein 1b in the fish liver and intestine compared to the controls. Meanwhile, adequate SePCH markedly enhanced the levels of immune factors (alkaline phosphatase, acid phosphatase, lysozyme, complement component 3, complement component 4 and immunoglobulin M) and innate immune-related genes (lysozyme, hepcidin, liver-expressed antimicrobial peptide 2, complement component 3 and complement component 4) in the fish liver and intestine compared to the controls. Adequate SePCH reduced the levels of pro-inflammatory cytokines (tumour necrosis factor-α, interleukin 8, interleukin 1β and interferon γ), while increasing transforming growth factor β1 levels at both transcriptional and protein levels in the liver and intestine. The mRNA expression levels of mitogen-activated protein kinase 13 (MAPK 13), MAPK14 and nuclear factor kappa B p65 were significantly reduced in the liver and intestine of fish fed with 0.60 and 1.20 g/Kg SePCH compared to the controls. Histological sections also demonstrated that 0.60 and 1.20 g/Kg SePCH significantly increased intestinal villus height and villus width compared to the controls. Furthermore, the mRNA expression levels of tight junction proteins (zonula occludens-1, zonula occludens-3, Claudin-1, Claudin-3, Claudin-5, Claudin-11, Claudin-23 and Claudin-34) and Mucin-17 were significantly upregulated in the intestinal epithelial cells of 0.60 and 1.20 g/Kg SePCH groups compared to the controls. In conclusion, these results found that 0.60 and 1.20 g/Kg dietary SePCH can not only improve growth, hematological parameters, selenium metabolism, antioxidant capacities, enhance immune responses and intestinal functions, but also alleviate inflammatory responses. This information can serve as a useful reference for formulating feeds for largemouth bass.

## 1 Introduction

As an essential trace element, selenium (Se) has garnered significant attention for its diverse biological functions, encompassing the enhancement of growth, promotion of normal development and provision of antioxidant and anti-inflammatory activities in both humans and animals (1–3). Selenium sources are classified into two categories based on their composition and origin: inorganic Se (such as Sodium Selenite) and organic Se compounds like selenomethionine (SeMet), selenoyeast, selenocysteine (SeCys) and methylselenocysteine (4, 5). It is widely recognized that Se fulfills its biological roles by becoming integrated into various selenoproteins, including selenocysteine lyase (SCLY), selenophosphate synthase 1 (SPS1), selenoprotein P (SEPP), selenoprotein T (SEPT), selenoprotein W (SEPW), selenoprotein H (SEPH), selenoprotein K (SEPK), 15kDa selenoprotein (SEP15) and glutathione peroxidase (GPx), among others (2, 5). Depending on the dosage at which it is administered, Se assumes distinct physiological functions in both humans and animals (2, 6). Numerous studies have substantiated that Se deficiency or excess can lead to growth retardation, oxidative stress, compromised immunity and dysfunction of the intestinal barrier in animals (1–3, 6). Consequently, organic Se is often included in diets to satisfy the physiological requirements for normal growth and health in animals due to its superior bioavailability and reduced toxicity (1, 2, 6, 7). Nevertheless, limited information is available regarding the utilization of organic Se from plants, which are widely available and more cost-effective, in animals.

Numerous studies have identified the potential of organic selenium (Se) to exhibit antioxidant and immune regulatory effects in both humans and animals (4, 8, 9). Excessive production or content of reactive oxygen species (ROS) during metabolic imbalances and immune stimulation and/or responses can lead to oxidative stress and disruption of physiological functions, which is often indicated by the antioxidant response in animals under ROS overload (10, 11). Selenium is involved in the formation of a range of selenoproteins (SPS1, SEP15, SEPT, SEPH, SEPK and GPx), where it exists in the form of SeCys and SeMet, playing pivotal antioxidant roles in animals (5). It is widely recognized that Se is an essential and integral component of selenoproteins (2). These selenoproteins play crucial roles in antioxidant defense by catalyzing the reduction of peroxides with the assistance of glutathione, thus counteracting peroxidation processes (8, 11). Studies have consistently demonstrated that Se deficiency can disrupt the antioxidant system, diminish the capacity to eliminate ROS and induce cellular damage in organisms (1, 12). Adequate Se levels can modulate intracellular redox status by regulating the expression and activities of redox-targeted proteins and enzymes, including superoxide dismutase (SOD), thioredoxin (Trx), catalase (CAT), GPx, glutathione reductase (GR), peroxiredoxin (Prx), glutathione S-transferase (GST), SEPH and SEPK, among others (4, 7). These regulatory mechanisms are primarily governed by the well-established Nrf2/Keap1 signaling pathway in human, mammals, and other animals (1, 13).

Furthermore, there exists a close relationship between the immune status of animals and hematological parameters, including white blood cells (WBC), neutrophils (NEU), red blood cells (RBC), hemoglobin (HGB), monocytes (MON), platelets (PLT), lymphocytes (LYM), mean corpuscular hemoglobin (MCH), mean corpuscular volume (MCV), mean corpuscular hemoglobin concentration (MCHC) and others (14, 15). Previous studies have highlighted that elevated levels of RBC, WBC, HGB and LYM can serve as indicators of improved immunity and can effectively enhance fish health by promoting the hematopoietic system through the provision of adequate nutrients (3, 16). Furthermore, immune capacities are intricately linked to antibacterial peptides, immune factors, immunoglobulins (Igs) and selenoproteins found in these immune tissues or organs of animals and fish (8, 17). Among these parameters, selenoproteins can enhance the immune process by regulating the synergistic interactions among these immune cells and inhibiting the expression and contents of inflammatory factors, include tumour necrosis factor-α (TNF-α), interleukins (ILs) and interferons (IFNs), etc. (10, 11). Previous research has proved Se deficiency can impair immune functions by reducing the production or contents of antibacterial compounds and immunoglobulins, as well as mRNA expression levels of antimicrobial peptides and selenoproteins (12, 17). Despite the numerous reports on the antioxidant and immune capacities mediated by sodium selenite and Se yeast, limited information is available concerning the comprehensive relationship between hematological parameters, antioxidant capacities, immune functions and dietary organic Se derived from plant sources in animals and fish species.

Moreover, previous studies have demonstrated Se deficiency can exacerbate inflammatory responses in humans and animals by modulating the levels of pro-inflammatory cytokines and anti-inflammatory cytokines, which include ILs, IFNs, TNF-α, transforming growth factor β1 (TGF-β1) and others (11, 12). Consequently, the overproduction of these inflammatory cytokines (e.g. TNF-α, IL-8, IL-1β and IFN-γ) can lead to cell damage and a decline in immune function in various tissues (10, 11). The content or production of these cytokines is primarily regulated by the p38 MAPK/NF-kB signaling transduction pathway in both humans and animals (4, 18, 19). However, these inflammatory processes can typically be mitigated by elevating the levels or contents of anti-inflammatory cytokines, such as TGF-β1 and IL-10 (11, 20). Furthermore, Se deficiency can severely impair the structure of mucosal cells and the physical barrier of the intestine, thereby limiting digestion and absorption and increasing the risk of disease in animals (21, 22). It is well-established that the intestinal integrity and functions are closely linked to the increased expression levels of tight junction proteins (TJs), including occludin (OCLN), zonula occludens (ZOs) and claudins (CLDNs) in the epithelial cells of the intestines in both humans and animals (23, 24). These proteins play a vital role in forming a protective gel that maintains the integrity and function of the intestinal barrier, accomplished through mucin-linked carbohydrates (22). Furthermore, as the key component of intestinal mucus, mucins (MUCs) are mainly produced by goblet cells to protect the epithelium from bacteria or hazardous materials (25). Consequently, MUCs serve critical structural and functional roles within the intestinal epithelial tissue and are essential for preserving intestinal health and immune function (26). Despite the fact that adequate inorganic Se and yeast-Se have been shown to alleviate inflammatory responses, protect the integrity of the intestinal structure and enhance intestinal barrier functions in animals (9, 24), there is limited information available about the effects of organic plant-derived Se on inflammatory responses and intestinal barrier functions in fish up to this point.

*Cardamine hupingshanensis*, a member of the cruciferous plant genus, is a distinctive organic Se source known for its elevated Se content across all its tissues (27–29). The primary bioactive compound within *C. hupingshanensis* is selenocysteine, which plays a pivotal role in selenoprotein synthesis, thereby contributing to a range of physiological functions (28, 29). Consequently, selenoprotein extracts from *C. hupingshanensis* (SePCH) constitute an innovative organic Se source endowed with robust antioxidant and immune-enhancing properties. However, there has been limited exploration into the use of SePCH as a dietary organic Se supplement in fish feed. As a carnivorous fish species, largemouth bass (*Micropterus salmoides*) has gained widespread cultivation in China owing to its rapid growth, robust adaptability and superior meat quality, thus establishing itself as one of the foremost commercial freshwater species in the country, with an output of 0.8 million tons in 2022 (30). While yeast-Se requirements have been studied in largemouth bass (1), the current knowledge landscape lacks insights into the SePCH requirements for this economically significant species in freshwater aquaculture. Consequently, this experiment is designed to evaluate the impacts of SePCH on largemouth bass, focusing on gene expression and protein production. Furthermore, we aim to elucidate the underlying mechanisms through which dietary SePCH influences intestinal barrier functions and the immune system, accomplished by analyzing hematological parameters, inflammation, oxidative stress and the transcription variations of TJs and MUCs. These findings provide valuable information for optimizing the formulation of compound feeds tailored to largemouth bass.

## 2 Materials and methods

The care and ethical treatment of the experimental animals strictly adhered to the “Guidance of the Care and Use of Laboratory Animals.” The research protocol involving the experimental subjects received approval from the National-Local Joint Engineering Laboratory of Aquatic Animal Genetic Breeding and Nutrition (Zhejiang).

### 2.1 Feed composition and experimental diets

The primary protein sources in these artificial diets were casein and fish meal, while rapeseed oil served as the principal lipid source. The base diet was supplemented with 0.00, 0.30, 0.60 and 1.20 g/Kg of SePCH to form four isonitrogenous and isoenergetic diets (see **Table 1** for details). The actual selenium contents are 0.37, 0.59, 0.84 and 1.30 mg/kg in these four different diets, respectively, measured with iCAPRQ ICP-MS (thermo, USA). All components underwent filtration using a 60-μm mesh and were blended according to the specifications provided in **Table 1**. Subsequently, these mixture underwent processing using a F-26 dual-screw extruder (Machinery Factory of South China University of Technology, Guangzhou, China), and made into pellets with 1.5 × 2.5 mm following the procedures outlined by Wu et al. (16) and Jia et al. (31). These pellets were dried at 35°C in one airflow drying oven for at least 24 hours and stored at -20°C until further use.

**Table 1:** Ingredient and proximate composition of basal diet (on dry weight basis).

Ingredients	Feed Diets (g/Kg)			
	0.00	0.30	0.60	1.20
Casein ^a^	350.00	350.00	350.00	350.00
Soybean meal ^b^	100.00	100.00	100.00	100.00
Gelatinized tapioca starch ^c^	100.00	100.00	100.00	100.00
Fish meal ^d^	200.00	200.00	200.00	200.00
Rapeseed oil ^e^	65.00	65.00	65.00	65.00
Microcrystalline cellulose ^f^	131.00	130.70	130.40	129.80
Mineral premix ^g^	20.00	20.00	20.00	20.00
Vitamin premix ^h^	10.00	10.00	10.00	10.00
Lecithin ^i^	20.00	20.00	20.00	20.00
Choline chloride ^j^	4.00	4.00	4.00	4.00
Selenoprotein extracts from *C. hupingshanensis* ^k^	0.00	0.30	0.60	1.20
Nutrient levels (g/kg dry matter basis)				
Crude protein	494.67	490.10	491.30	482.31
Crude lipid	114.7	117.7	118.0	118.0
Moisture	36.86	33.86	35.73	37.45
Crude Ash	59.91	60.23	60.29	60.41

a Casein, obtained from Gansu Hualing Dairy Co., Ltd., Lanzhou, China; crude protein 80.56%.

b Soybean meal, obtained from Ningbo Food Co., Ltd., Zhejiang, China.

c Gelatinized tapioca starch, obtained from Xinxin biochemical technology Co., Ltd., Zhejiang, China.

d Fish meal, Pesquera Diamante S.A. crude protein 66.7%; crude lipid 9.8%.

e Rapeseed oil, produced using oil press.

f Microcrystalline cellulose, obtained from Sinopharm Chemical Reagent Co., Ltd., Shanghai, China.

g Mineral premix (mg/kg) Mineral mixture: KI 0.4mg, CoCl_2_-6H_2_O 52 mg, CuSO_4_-5H_2_O 16 mg, FeSO_4_-7H_2_O 200 mg, ZnSO_4_-H_2_O 280 mg, MnSO_4_-H_2_O 45 mg, MgSO_4_-7H_2_O 1200 mg, Ca(H_2_PO_4_)_2_ 12000 mg, NaCl 60 mg.

h Vitamin premix (mg/kg) Vitamin premix: vitamin A, 20 mg; vitamin D3, 3 mg; vitamin C, 300 mg; vitamin E, 300 mg; thiamin, 20 mg; riboflavin, 10 mg; pyridoxine HCl, 20 mg; vitamin B12, 0.2 mg; vitamin K3, 5 mg; inositol, 1000 mg; pantothenic acid, 30 mg; folic acid, 3 mg; niacin acid, 50 mg; biotin, 1 mg.

i lecithin, Jiangsu Yuanshengyuan Biological Engineering Co., Ltd., Nanjing, China.

j Choline chloride, obtained from Xinxin biotechnology Co., Ltd., Shandong, China.

k Purchased from Hubei Shengxi Biotechnology Co Ltd: The selenium content in the Selenium-enriched C. hupingshanensis was 764 mg/kg.

### 2.2 Fish and feeding trial

The feeding trial took place at the Aquatic animal culture center, Huzhou University. Healthy juvenile largemouth bass were procured from Zhejiang Deqing Longshengli Aquatic Hatchery (Huzhou, China) and acclimated in a recirculating aquaculture tanks for a period of two weeks prior to the experiment. During this acclimation period, they were provided with a basal diet devoid of SePCH. A total of 300 juvenile largemouth bass, initially weighing (5.04 ± 0.02)g, were selected and randomly allocated to 12 aquaculture tanks. There were 4 different experimental groups in these tanks, each with 3 replicates. Within each replicate, 25 fish were reared for a duration of 60 days. The daily feed intake during the culture period was maintained at 3% of the total fish weight and was administered at 8:00 and 17:00, respectively. Tank water was exchanged every other day, and thorough suctioning was performed to ensure water quality. Throughout the experimental period, water temperature was mainly stayed at 26.5-28.5°C, with natural lighting conditions and dissolved oxygen levels exceeding 5.8 mg/L.

### 2.3 Sample collection and detection of growth performances

After a 24-hour fasting period, fish from these 12 tanks were counted at the end of feeding trial. Subsequently, they were weighed to evaluate weight gain (WG) and daily growth rate (DGR) after being anesthetized with tricaine methane sulfonate (100 ppm) (MS-222, Sigma, St. Louis, MO, USA). Samples were then collected on ice for further analyses. Blood for hematological analysis was obtained from the caudal tail veins of fifteen fish randomly selected from each tank. Approximately 0.3 mL of blood was selected in 2 ml heparinized Eppendorf tubes. Liver and intestine specimens were also collected and promptly flash-frozen in liquid nitrogen and then deposited at -80 °C for the next measurements and analysis of relative enzyme activity and gene transcription variations.

### 2.4 Hematological analyses

TEK 8500VET automatic blood analyzer provided by Jiangxi Tekang Technology (Nanchang, China) was employed for hematological analysis, which encompassed assessments of total cell counts and various cell categories. Parameters examined included counts of WBC, RBC, HGB, PLT, MCV, MCH, MCHC, MON, LYM and NEU. Fifteen duplicate blood samples from each group were subjected to analysis

### 2.5 Measurements of antioxidant enzyme activities and immunological parameters

To assess antioxidant enzyme activities’ variation in these fish liver and intestine, frozen tissue samples were initially pulverized using liquid nitrogen. Subsequently, the samples were homogenized with a 0.9% NaCl solution at a volume ratio of 1:9 (w/v) at 4°C. Following homogenization, the suspensions were subjected to centrifugation at 3000 rpm for 20 minutes at 4°C, and the resulting supernatants were collected. Coomassie Brilliant Blue technique was used to determine the protein concentrations of these supernatants. Activities or contents of SOD, CAT, GPx, GST, GR, glutathione (GSH), malondialdehyde (MDA), hydrogen peroxide (H_2_O_2_), total antioxidant capacity (T-AOC) and Kelch-like ECH-associated protein 1a (Keap1a) were determined with commercially available kits in accordance with these corresponding instructions provided by Jiancheng Bioengineering (Jiancheng, Nanjing, China). The activities or the levels of lysozyme, complement component 3 (C3), C4, acid phosphatase (ACP), IgM, alkaline phosphatase (ALP), TNF-α, IL-1β, IL-8, IFN-γ and TGF-β1 were also measured using diagnostic assay kits following these corresponding instructions provided by Henyuan Biotech (Shanghai, China). Each measurement was carried out in triplicate.

### 2.6 Measurements of genes expression

The total RNA were extracted from fish liver and intestine samples using the TRIzol reagent (Invitrogen, USA). The concentration and integrity of these RNA samples were detected through spectrophotometry and electrophoresis following the procedures outlined by Wu et al. (32). After treatment with DNase I treatment (Takara, Dalian, China), 10 µg of total RNA samples were reverse transcribed with SuperScript™ II RT (Takara, Dalian, China) in 75 µL reactions. Primers for quantitative real-time PCR were designed according to largemouth bass genes (**Table 2**). Primer synthesis was carried out by Biosune Co. (Shanghai, China). β-actin was served as the internal reference. Quantitative PCR (qPCR) assays were conducted following the methods described by Wu et al. (16) and Yang et al. (33). Each analysis was performed carried out in at least triplicate.

**Table 2:** Primer sequences for real-time PCR analysis.

Gene	Forward (5′− 3′)	Reverse (5′− 3′)	Reference
SCLY	GTGCCGCCTGCCACTCAAA	TCCTGCCCACGCTCAACCT	XM_038716707.1
SPS1	CGACATCACAGGGTTTGGC	TTGGCTAGGACTGGGAGGTTA	XM_038705397.1
SEP15	GGAGTGAAAAGCCGAAGATGT	CTTCAGCGATGTTCCCGTTA	XM_038718375.1
SEPT2	AGAGCCTGGACCTGGAGT	TCGTTTAGCGTGACTTCG	XM_038699855.1
SEPH	AGGCTGATGAGGAGAAAG	GCATTACGCCCATACACT	XM_038699684.1
SEPP	CTCACACACCACATTTCAC	ATCAGCTTTCTCTTTGCAC	XM_038702803.1
SEPK	TGCTTCGTCACGCTTCACT	TCATCCTCCTCCACCCATT	XM_038723206.1
Cu/Zn-SOD	TGAGCAGGAGGGCGATTC	GCACTGATGCACCCATTTGTA	XM_038708943.1
Mn-SOD	CAGGGATCTACAGGTCTCATT	ACGCTCGCTCACATTCTC	XM_038727054.1
CAT	ACCTATTGCTGTCCGCTTCTC	TCCCAGTTGCCCTCCTCA	XM_038704976.1
GPx1	TTTGGAGTCCCGTCTGTA	CTGCCTCAATGTCAATGGT	XM_038697220.1
GPx3	CCCTCCAGTTGGAAACGA	ACTTGGGTGCCACCTCAT	XM_038699914.1
GR	ATCACGAGCAGGAAGAGTCAG	CATCTCATCACAGCCCAAGC	XM_038700350.1
GST	GAGCCCATCAGAACACCC	ACCCAAATAGCACCCAAC	XM_038729946.1
Keap1a	GTGGTGGGAAGACTTATTG	TCCAGGTGCTTAGTGAGG	XM_038728593.1
Keap1b	CCTTACTCCAGGCTGTCCG	GAAATTACTTTGGTGGGTTTGT	XM_038713665.1
Nrf2	CAAAGACAAGCGTAAGAAGC	CAGGCAGATTGATAATCATAGA	XM_038720536.1
C3	CCACTATGCCACGAGAAC	GAGCGTAATACAGCGACAC	XM_038714039.1
C4	TGGTGGTCGTCCTGCGTC	CCCTCCTGGTTGGTGGTG	XM_038711699.1
lysozyme	CTCTCATTGCTGCCATCA	TGTGTCCACCTCCGTTTG	XM_038713808.1
HEPC	CTCTGCCGTCCCATTCAC	GCATCATCCACGATTCCATT	XM_038710826.1
LEAP-2	AAGGAAAGCAGCAGTAGCG	CTGCCTTCTGGTCAGAGTTG	XM_038731861.1
TGF-β1	CCCGCTTCATCACTAATA	GTTGGAAACCCTTCTCAT	XM_038693206.1
IL-10	CCAGCAGCATCATTACCA	CAGAACCAGGACGGACAG	XM_038696252.1
IL-1β	CGACCGCAGTAAGAAAGA	TCGATGTACCTCGAAAGT	XM_038733429.1
IL-8	TGGCACTCCTGGTCATCC	GCACCTCCACCTGTCCTAT	XM_038713529.1
TNF-α	CAACGGCAAGTGTCAAACCC	TCTTGTCCTGAGCCCTTGGTAT	XM_038723994.1
IFN-γ	GAGTTGCTTTGGCGTTTG	TGTTGATGCTCCTGGTGA	XM_038709291.1
NF-κB p65	GCACAGGACGAGGATGGA	CAGGCAGGGCAGAGACAA	XM_038699792.1
MAPK13	GATGCTGGTTCTGGATGG	TAGGCTCAGGAAAGTCGT	XM_038723459.1
MAPK14	CCACGTTCAGTTCCTTATCTAC	CTCGCAGTCTTCATTCACAG	XM_038696748.1
ZO-1	TTCTAACGGTGGTGTCCTG	TTGTCAAGTGGTGGCAAG	XM_038701018.1
ZO-3	GGACCCCCAGACTTTGTA	AGAGCGAGCCTGTTGTAA	XM_038716281.1
OCLN	ATTCCTGGTTGTCATCGC	AGAGGCTCATCCCAGTAGA	XM_038715419.1
CLDN-1	CTCTGTGGCTGTGGGAACT	GAACATGGTGTCGCTGTAAGT	XM_038713307.1
CLDN-3	CCCGTGCCCTCACCGTTAT	TTGGATGCCTCGTCGTCA	XM_038693400.1
CLDN-11	CCAAGAACAAACGCACCA	TAGAGGGAGAAGCCGAAT	XM_038729166.1
CLDN-5	AGGTGCCCGCATCCCAGAA	AGGGACAGGAGCAGCACAGC	XM_038704228.1
CLDN-23	TGTCTTCACTGCCGCTAT	CCCTGACCTTTCACTCCT	XM_038729173.1
CLDN-34	GGAACGTCTACTTTGGGA	GGAACTTGATGGTCTGAT	XM_038715311.1
MUC-2	CTGTATGCCCAGAAGATAAGC	TGGTAGCACGTTCCCAAA	XM_038706114.1
MUC-5AC	ATGGACAACAGACTGATCTTCG	GGTTATCGTCGTCGTAGGG	XM_038731574.1
MUC-17	ATGAAACCATAACCGTAC	TATCACCACAATGAGCAG	XM_038725272.1
β-actin	TCCTGCGTCTTGACTTGG	GATTTCCCTTTCGGCTGT	XM_038695351.1

SCLY, selenocysteine lyase; SPS1, selenophosphate synthase 1; SEP15, 15 kDa selenoprotein; SEPT2, selenoprotein T2; SEPH, selenoprotein H; SEPP, selenoprotein P; SEPK, selenoprotein K; Cu/Zn-SOD, Cu/Zn-Superoxide dismutase; Mn-SOD, Mn-Superoxide dismutase; CAT, catalase; GPx3, glutathione peroxidase 3; GPx1, glutathione peroxidase 1; GR, glutathione reductase; GST, glutathione S-transferase; Keap1a, kelch-like ECH-associated protein 1a; Keap1b, kelch-like ECH-associated protein 1b; Nrf2, NF-E2-related factor 2; C3, complement component 3; C4, complement component 4; lysozyme; HEPC, hepcidin; LEAP-2, liver-expressed antimicrobial peptide 2; IL-10, interleukin 10; TGF-β1, transforming growth factor β1; IL-1β, interleukin-1β; IL-8, interleukin 8; TNF-α, tumour necrosis factor-α; IFN-γ, interferon-γ; NF-κB, nuclear factorkappa B; MAPK13, mitogen-activated protein kinase 13; MAPK14, mitogen-activated protein kinase 14; ZO-1, zonula occludens-1; ZO-3, zonula occludens-3; OCLN, Occludin; CLDN-1, Claudin-1; CLDN-3, Claudin-3; CLDN-11, Claudin-11; CLDN-5, Claudin-5; CLDN-23, Claudin-23; CLDN-34, Claudin-34; MUC-2, Mucin-2; MUC-5AC, Mucin-5AC; MUC-17, Mucin-17.

### 2.7 Measurements of histomorphometry

Posterior intestine samples were firstly rinsed with 0.6% saline and then fixed in 4% paraformaldehyde for 48 hours. Subsequently, the samples underwent dehydration in a series of ethanol solutions with increasing concentrations, clearing with xylene and embedding in paraffin wax. The hematoxylin and eosin (H&E) staining method was used to generate and stain histological sections. Photographic documentation of the intestine was carried out by capturing micrographs at a final magnification of 100× using a digital camera. These acquired images were then subjected to analysis using K-Viewe 1.5 software (https://kv.kintoneapp.com/en/user/, accessed on 16 June 2023) to quantify villus height, villi width, muscle thickness and crypt depth. All measurements were performed with a minimum of three replicates.

### 2.8 Statistical analyses

The following equations were used to calculate animal-specific parameters:


$$\begin{array}{c} \text{Weight gain }(\text{WG},\%)=[(\text{final body weight }(\text{FBW})\\ -\text{ initial body weight }(\text{IBW})\text{/initial body weight }(\text{IBW})]\times 100. \end{array}$$

$$\begin{array}{c} \text{Daily growth rate }(\text{DGR},\%)=[(\text{final body weight }(\text{FBW})\\ -\text{ initial body weight }(\text{IBW})\text{/initial body weight }(\text{IBW})\\ \;*\;60\;\text{days}]\times 100. \end{array}$$

The results derived from the analysis were presented as mean ± standard deviation (SD). To compare these various groups, a one-way analysis of variance (ANOVA) was performed with SPSS 22.0 software (IBM, Chicago, IL, USA). For additional comparisons among the diverse dietary treatments, Duncan’s multiple range test was employed with a multiple comparison test. Additionally, to investigate any significant linear or quadratic effects, orthogonal polynomial comparisons were conducted. *p<* 0.05 was utilized to determine the presence of statistical differences.

## 3 Results

### 3.1 Growth performance and hematological indices

**Figure 1:**
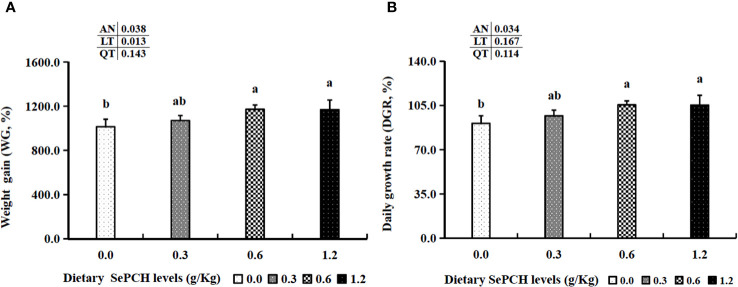
Effect of dietary SePCH on weight gain (%) **(A)** and daily growth rate (%) **(B)** of juvenile largemouth bass. Bars with different letters indicate significant differences (*p*< 0.05), while that with the same letter or no letter superscripts indicate no significant differences (*p* > 0.05), AN, ANOVA; LT, linear trend; QT, quadratic trend. The same as the following Figures.

Following an 8-week cultivation period, the dietary inclusion of SePCH exhibited a beneficial impact on the growth parameters of largemouth bass. Evaluation of the growth parameters revealed that supplementation with 0.60 and 1.20 g/Kg SePCH markedly increased both WG and DGR compared to the control groups (0.00 g/Kg SePCH) (*p*< 0.05) (**Figure 1**). Furthermore, several hematological indicators showed significant improvements in the 0.60 and 1.20 g/Kg SePCH groups compared to the SePCH deficient groups or control groups (*p<* 0.05). Specifically, counts or levels of WBC, MCV, MCH and MON were markedly enhanced in 0.60 and 1.20 g/Kg SePCH groups compared to the control groups (*p*< 0.05). Additionally, LYM counts notably increased in 0.30, 0.60 and 1.20 g/Kg SePCH groups compared to the controls (*p*< 0.05). As the levels of SePCH supplementation increased, RBC, HGB and PLT levels also increased significantly, reaching their highest levels in the 0.60 g/Kg SePCH groups (*p*< 0.05), although no marked difference was observed between the 0.60 and 1.20 g/Kg SePCH groups. Moreover, no significant changes were obtained in terms of MCHC and NEU values across these four experimental groups (**Table 3**).

**Table 3:** Haematological parameters of juvenile largemouth bass fed diet trials.

	Supplemental levels of SePCH (g/Kg)						
Parameters	0.00	0.30	0.60	1.20	AN	LT	QT
WBC (10^9^/L)	158.93 ± 2.27 ^b^	159.33 ± 1.70 ^b^	170.54 ± 1.56 ^a^	167.94 ± 5.56 ^a^	0.004	0.017	0.150
RBC (10^12^/L)	2.76 ± 0.08 ^c^	3.01 ± 0.05 ^bc^	3.32 ± 0.05 ^a^	3.08 ± 0.23 ^ab^	0.001	0.005	0.023
HGB (g/L)	93.67 ± 2.08 ^c^	105.33 ± 1.53 ^bc^	114.33 ± 5.86 ^a^	112.33 ± 4.16 ^ab^	0.001	0.004	0.004
PLT (10^9^/L)	105.00 ± 3.61 ^b^	125.67 ± 7.64 ^ab^	140.33 ± 15.04 ^a^	133.33 ± 21.20 ^ab^	0.620	0.360	0.640
MCV (fL)	182.53 ± 2.21 ^b^	186.13 ± 1.70 ^b^	199.57 ± 3.51 ^a^	199.90 ± 3.36 ^a^	0.000	0.001	0.056
MCH (pg)	33.20 ± 1.23 ^b^	34.67 ± 0.95 ^ab^	35.80 ± 0.46 ^a^	36.30 ± 0.70 ^a^	0.003	0.001	0.419
MCHC (pg)	186.00 ± 1.00	187.67 ± 3.06	188.00 ± 1.73	188.00 ± 2.00	0.613	0.287	0.393
NEU (10^9^/L)	6.29 ± 0.74	6.32 ± 0.66	6.49 ± 0.82	6.13 ± 0.58	0.941	0.774	0.614
LYM (10^9^/L)	135.90 ± 7.12 ^b^	145.00 ± 2.27 ^a^	149.83 ± 1.20 ^a^	146.45 ± 4.31 ^a^	0.018	0.300	0.046
MON (10^9^/L)	4.53 ± 0.38 ^b^	4.60 ± 0.10 ^b^	5.50 ± 0.26 ^a^	5.10 ± 0.10 ^a^	0.004	0.057	0.660

^a–c^ Values in the same row with different letters indicate signiﬁcant differences (p< 0.05), while that with the same letter or no letter superscripts indicate no significant differences (p > 0.05). AN, ANOVA; LT, linear trend; QT, quadratic trend. The same as the following Tables.

### 3.2 Analyses of antioxidants and oxidants in fish liver and intestine

Significant variations were observed in the antioxidative indices among the different test groups of fish. In comparison to the 0.00 and 0.30 g/Kg groups, the activities of T-SOD, CAT, GPx and GR were markedly higher in the fish liver of 0.60 and 1.20 g/Kg SePCH groups (*p*< 0.05), with no notable difference in 0.60 and 1.20 g/Kg groups. Conversely, MDA contents exhibited a significant reduction in the 0.60 and 1.20 g/Kg SePCH groups (*p*< 0.05) compared to the controls (*p<* 0.05). The contents of H_2_O_2_ showed a significant decrease in the 0.60 and 1.20 g/Kg SePCH groups compared to the 0.00 and 0.30 g/Kg SePCH groups (*p*< 0.05), with no significant difference between 0.00 and 0.30 g/Kg SePCH groups. Compared to the 0.00 and 0.30 g/Kg groups, the contents of Keap1a were markedly decreased in the 0.60 and 1.20 g/Kg SePCH groups (*p*< 0.05), with no significant difference between the two groups. However, the contents of T-AOC and GSH were markedly increased with the increase in SePCH addition, reached the highest levels in the 0.60 g/Kg SePCH groups (*p*< 0.05), and no marked difference was obtained between the 0.60 g/Kg and 1.20 g/Kg SePCH groups. However, GST activities presented no notable difference among all these four fish groups (**Table 4**).

**Table 4:** Effects of dietary SePCH on the antioxidative and oxidative indices in the liver and intestine of largemouth bass.

	Supplemental levels of SePCH (g/Kg)						
Parameters	0.00	0.30	0.60	1.20	AN	LT	QT
Liver							
T-SOD (U/mg prot)	9.27 ± 1.59 ^c^	10.82 ± 0.35 ^bc^	13.98 ± 0.78 ^a^	12.44 ± 0.61 ^ab^	0.002	0.029	0.007
CAT (U/mg prot)	5.47 ± 1.32 ^b^	7.11 ± 0.99 ^b^	17.53 ± 0.78 ^a^	15.61 ± 0.66 ^a^	0.000	0.002	0.310
GPx (U/mg prot)	45.81 ± 2.17 ^b^	49.84 ± 1.47 ^b^	126.98 ± 7.39 ^a^	125.61 ± 4.27 ^a^	0.000	0.001	0.111
GR (U/g prot)	11.25 ± 1.53 ^b^	12.56 ± 0.98 ^b^	15.37 ± 1.78 ^a^	15.17 ± 0.71 ^a^	0.013	0.007	0.090
GST (U/mg prot)	206.93 ± 1.72	205.20 ± 2.99	202.33 ± 1.00	204.05 ± 4.98	0.372	0.258	0.199
GSH (μmol/g prot)	107.36 ± 1.09 ^b^	112.52 ± 5.19 ^ab^	118.96 ± 0.65 ^a^	118.09 ± 4.53 ^a^	0.012	0.009	0.380
T-AOC (mmol/g prot)	36.23 ± 1.45 ^c^	40.88 ± 0.62 ^a^	42.22 ± 0.59 ^a^	38.68 ± 0.36 ^b^	0.000	0.435	0.000
MDA (mmol/g prot)	1.25 ± 0.07 ^a^	1.18 ± 0.05 ^ab^	1.05 ± 0.08 ^b^	1.10 ± 0.60 ^b^	0.320	0.045	0.045
Keap1a (ng/l)	154.57 ± 9.96 ^a^	143.89 ± 2.67 ^a^	116.97 ± 6.45 ^b^	115.21 ± 14.70 ^b^	0.002	0.001	0.085
H_2_O_2_ (mmol/g prot)	30.60 ± 0.98 ^a^	27.29 ± 0.97 ^b^	23.18 ± 0.42 ^c^	21.68 ± 0.31 ^c^	0.000	0.000	0.001
Intestine							
T-SOD (U/mg prot)	11.68 ± 1.05 ^c^	13.17 ± 1.16 ^bc^	14.92 ± 0.05 ^ab^	16.53 ± 0.88 ^a^	0.001	0.000	0.248
CAT (U/mg prot)	32.39 ± 4.65 ^c^	36.75 ± 6.48 ^bc^	47.81 ± 0.85 ^a^	44.97 ± 6.58 ^ab^	0.022	0.022	0.079
GPx (U/mg prot)	1.92 ± 0.14 ^b^	2.00 ± 0.30 ^b^	2.90 ± 0.17 ^a^	3.01 ± 0.30 ^a^	0.000	0.000	0.161
GR (U/g prot)	19.03 ± 1.88 ^c^	31.76 ± 1.87 ^b^	35.20 ± 1.86 ^ab^	37.18 ± 1.77 ^a^	0.000	0.001	0.000
GST (U/mg prot)	38.73 ± 1.15 ^c^	41.26 ± 0.67 ^b^	42.19 ± 0.46 ^ab^	43.54 ± 1.21 ^a^	0.001	0.000	0.630
GSH (μmol/g prot)	62.04 ± 1.19 ^b^	65.57 ± 3.55 ^ab^	69.24 ± 1.42 ^a^	66.57 ± 0.72 ^a^	0.016	0.086	0.008
T-AOC (mmol/g prot)	16.35 ± 0.43 ^b^	18.50 ± 3.41 ^b^	26.73 ± 1.59 ^a^	24.67 ± 1.02 ^a^	0.001	0.007	0.310
MDA (mmol/g prot)	1.97 ± 0.02 ^a^	1.35 ± 0.02 ^b^	1.36 ± 0.01 ^b^	1.32 ± 0.01 ^b^	0.000	0.009	0.001
Keap1a (ng/l)	160.13 ± 21.18 ^a^	134.49 ± 16.06 ^ab^	101.58 ± 3.70 ^c^	110.66 ± 6.02 ^bc^	0.003	0.009	0.010
H_2_O_2_ (mmol/g prot)	10.90 ± 0.16 ^a^	10.76 ± 0.11 ^a^	9.48 ± 0.22 ^b^	9.23 ± 0.23 ^b^	0.000	0.000	0.203

Activities of T-SOD and CAT were markedly enhanced in the fish intestine of 0.60 and 1.20 g/Kg SePCH groups in comparison with the controls (*p*< 0.05), although there were no notable difference between these two groups. Meanwhile, GPx activities and T-AOC contents significantly increased in the 0.60 and 1.20 g/Kg SePCH groups compared to the 0.00 and 0.30 g/Kg SePCH groups (*p*< 0.05), although no marked difference was observed between the 0.60 and 1.20 g/Kg SePCH groups. The activities of GR and GST exhibited a progressive rise that was positively linked to the doses of SePCH (*p*< 0.05), reaching its maximum in the 1.20 g/Kg SePCH groups with no significant difference between the 0.60 and 1.20 g/Kg SePCH groups. Compared to the 0.00 g/Kg SePCH groups, the contents of GSH significantly increased in the 0.60 and 1.20 g/Kg SePCH groups (*p*< 0.05), with no marked difference between 0.60 and 1.20 g/Kg groups. However, in comparison to the 0.00 g/Kg SePCH groups, the contents of MDA were significantly decreased in the 0.30, 0.60 and 1.20 g/Kg SePCH groups (*p*< 0.05), and there were no marked difference between these three groups. Meanwhile, the 0.60 g/Kg SePCH groups exhibited the lowest Keap1a levels (*p*< 0.05) compared to the 0.00 and 0.30 g/Kg SePCH groups, although there was no notable difference between the 0.60 and 1.20 g/Kg SePCH groups. H_2_O_2_ contents in the intestine of 0.60 and 1.20 g/Kg SePCH groups were markedly lowed in comparison with those in the 0.00 and 0.30 g/Kg SePCH groups (**Table 4**).

### 3.3 Analysis of immune parameters in fish liver and intestine

Lysozyme activities and IgM contents in the fish liver of 0.60 and 1.20 g/Kg SePCH groups were significantly heightened in comparison with the controls (*p*< 0.05), with no noticeable difference compared to the 0.30 g/Kg SePCH groups (**Table 5**). The activities of ACP reached the maximum in the 1.20 g/Kg SePCH groups and noticeably higher than those in the SePCH deficient groups (*p*< 0.05). The activities of ALP were significantly higher in the 0.60 g/Kg SePCH groups than these in other three groups (*p*< 0.05). Meanwhile, the levels of C3 and C4 in the 0.60 and 1.20 g/Kg SePCH groups were markedly higher than those in the 0.00 and 0.30 g/Kg SePCH groups (*p*< 0.05), although no marked difference was shown between the 0.60 and 1.20 g/Kg SePCH groups.

**Table 5:** Effect of dietary SePCH on the immune parameters and the anti-inflammatory and pro-inflammatory cytokines in the liver of juvenile largemouth bass.

	Supplemental levels of SePCH (g/Kg)						
Parameters	0.00	0.30	0.60	1.20	AN	LT	QT
lysozyme (U/mg prot)	47.57 ± 4.07 ^b^	51.97 ± 1.39 ^ab^	59.70 ± 4.86 ^a^	57.73 ± 4.48 ^a^	0.020	0.019	0.054
ACP (U/g prot)	4.41 ± 0.16 ^c^	6.62 ± 0.31 ^ab^	6.02 ± 0.05 ^b^	7.01 ± 0.75 ^a^	0.000	0.006	0.116
ALP (U/g prot)	124.87 ± 2.81 ^c^	125.17 ± 1.08 ^c^	150.93 ± 1.43 ^a^	133.90 ± 1.14 ^b^	0.000	0.173	0.020
C3 (μg/ml)	57.27 ± 0.72 ^b^	57.94 ± 5.78 ^b^	61.65 ± 1.44 ^a^	66.85 ± 2.37 ^a^	0.023	0.001	0.656
C4 (μg/ml)	59.58 ± 1.50 ^b^	69.47 ± 3.18 ^b^	88.36 ± 1.64 ^a^	90.80 ± 14.45 ^a^	0.002	0.001	0.074
IgM (μg/ml)	269.18 ± 15.30 ^b^	284.56 ± 9.04 ^ab^	299.70 ± 7.03 ^a^	300.49 ± 4.59 ^a^	0.014	0.006	0.054
TGF-β1 (U/mg prot)	3.67 ± 0.52 ^b^	3.84 ± 0.54 ^b^	7.41 ± 0.40 ^a^	7.06 ± 0.26 ^a^	0.000	0.002	0.105
IL-1β (U/g prot)	18.03 ± 1.82 ^a^	16.94 ± 1.19 ^ab^	14.12 ± 0.70 ^bc^	13.10 ± 1.90 ^c^	0.011	0.001	0.333
TNF-α (U/mg prot)	31.03 ± 3.13 ^a^	30.61 ± 2.03 ^a^	24.01 ± 2.44 ^b^	22.92 ± 3.52 ^b^	0.014	0.004	0.465
IFN-γ (ng/L)	41.91 ± 5.14 ^a^	37.25 ± 1.85 ^b^	31.43 ± 3.33 ^b^	31.86 ± 4.90 ^b^	0.038	0.015	0.094
IL-8 (pg/L)	31.89 ± 0.30 ^a^	28.17 ± 1.76 ^ab^	21.96 ± 5.37 ^b^	23.91 ± 4.71 ^b^	0.042	0.035	0.068
IL-12 (ng/L)	24.06 ± 0.58 ^a^	22.93 ± 1.11 ^ab^	21.61 ± 0.71 ^b^	21.61 ± 0.47 ^b^	0.011	0.005	0.050

Regarding anti-inflammatory factors, the levels of TGF-β1 displayed a significant increase in the fish liver of 0.60 and 1.20 g/Kg SePCH groups compared to the 0.00 and 0.30 g/Kg SePCH groups (*p*< 0.05), although no notable difference was presented between the 0.60 and 1.20 g/Kg SePCH groups (**Table 5**). However, concerning pro-inflammatory factors, the contents of IL-1β, IL-8 and IL-12 significantly decreased in the 0.60 and 1.20 g/Kg SePCH groups compared to the 0.00 g/Kg SePCH groups (*p*< 0.05), although there were no noticeable differences in 0.00 and 0.30 g/Kg SePCH groups. The levels of TNF-α showed a significant reduction in the 0.60 and 1.20 g/Kg SePCH groups compared to the 0.00 and 0.30 g/Kg SePCH groups (*p*< 0.05), although no marked difference was shown between the 0.60 and 1.20 g/Kg SePCH groups. Meanwhile, compared to the control groups, the contents of IFN-γ were noticeably reduced in the 0.30, 0.60 and 1.20 g/Kg SePCH groups (*p*< 0.05), and no marked differences were observed among those three groups.

Immunological parameters were also evaluated in the intestinal tissues of our experimental fish (**Table 6**). In comparison to the 0.00 and 0.30 g/Kg SePCH groups, the activities of lysozyme and IgM showed a noticeable increase in the 0.60 and 1.20 g/Kg SePCH groups (*p*< 0.05), and no marked difference was presented between the 0.60 and 1.20 g/Kg groups. Meanwhile, compared to the 0.00 g/Kg SePCH groups, the activities of ACP and ALP were markedly enhanced in the 0.60 and 1.20 g/Kg SePCH groups (*p*< 0.05), although no marked differences were obtained among the 0.30, 0.60 and 1.20 g/Kg SePCH groups. The contents of C3 in the intestine were markedly elevated in the 0.30 g/Kg to 1.20 g/Kg SePCH groups in comparison with the controls (*p*< 0.05). At the SePCH additive levels of 1.20 g/Kg, the levels of C4 were notably higher than these in other three groups (*p*< 0.05).

**Table 6:** Effect of dietary SePCH on the immune parameters and the anti-inflammatory and pro-inflammatory cytokines in the intestine of juvenile largemouth bass.

	**Supplemental levels of SePCH (g/Kg)**						
Parameters	0.00	0.30	0.60	1.20	AN	LT	QT
lysozyme (U/mg prot)	236.01 ± 17.82 ^b^	254.02 ± 27.10 ^b^	369.77 ± 27.10 ^a^	349.20 ± 20.42 ^a^	0.920	0.004	0.059
ACP (U/g prot)	21.06 ± 1.28 ^b^	22.05 ± 0.43 ^ab^	23.58 ± 0.29 ^a^	23.14 ± 0.77 ^a^	0.017	0.020	0.040
ALP (U/g prot)	3.01 ± 0.15 ^b^	3.15 ± 0.01 ^ab^	3.23 ± 0.06 ^a^	3.28 ± 0.07 ^a^	0.026	0.005	0.142
C3 (μg/ml)	85.15 ± 11.57 ^b^	127.14 ± 2.18 ^a^	142.51 ± 16.38 ^a^	142.60 ± 5.32 ^a^	0.000	0.004	0.001
C4 (μg/ml)	17.48 ± 1.53 ^d^	21.15 ± 1.00 ^c^	25.48 ± 1.53 ^b^	29.15 ± 1.00 ^a^	0.000	0.000	0.053
IgM (μg/ml)	177.02 ± 3.54 ^b^	179.01 ± 4.03 ^b^	219.90 ± 8.72 ^a^	222.39 ± 5.24 ^a^	0.000	0.000	0.185
TGF-β1 (U/mg prot)	39.18 ± 1.33 ^c^	45.69 ± 2.78 ^b^	57.67 ± 0.65 ^a^	56.80 ± 1.97 ^a^	0.000	0.001	0.002
IL-1β (U/g prot)	82.96 ± 2.10 ^a^	41.65 ± 1.99 ^b^	36.44 ± 1.70 ^c^	35.64 ± 0.70 ^c^	0.000	0.005	0.000
TNF-α (U/mg prot)	70.36 ± 1.93 ^a^	61.64 ± 4.44 ^ab^	49.85 ± 6.11 ^b^	47.54 ± 8.67 ^b^	0.004	0.001	0.690
IFN-γ (ng/L)	90.20 ± 5.95 ^a^	88.24 ± 1.48 ^a^	55.88 ± 7.78 ^b^	62.50 ± 5.84 ^b^	0.000	0.006	0.087
IL-8 (pg/L)	63.91 ± 3.98 ^a^	58.39 ± 3.20 ^ab^	50.80 ± 5.37 ^b^	51.15 ± 4.04 ^b^	0.836	0.007	0.630
IL-12 (ng/L)	38.90 ± 2.32 ^a^	32.18 ± 3.57 ^b^	27.96 ± 3.49 ^b^	27.84 ± 1.60 ^b^	0.005	0.005	0.014

Regarding intestinal anti-inflammatory factors, the levels of TGF-β1 in the 0.60 and 1.20 g/Kg SePCH groups were noticeably higher than those in the 0.00 and 0.30 g/Kg SePCH groups (*p*< 0.05), and no difference was shown in the 0.60 and 1.20 g/Kg groups (**Table 6**). However, in comparison to the controls, the contents of IL-8 and TNF-α were markedly decreased in the 0.60 and 1.20 g/Kg SePCH groups in comparison to the controls (*p*< 0.05), and no marked difference was presented among the 0.30, 0.60 and 1.20 g/Kg SePCH groups. The contents of IFN-γ and IL-1β in the 0.60 and 1.20 g/Kg SePCH groups exhibited significant reductions compared to the 0.00 and 0.30 g/Kg SePCH groups (*p*< 0.05), with no notable difference between the 0.60 and 1.20 g/Kg groups. Similarly, compared to the controls, the contents of IL-12 significantly decreased with SePCH addition from 0.30 g/Kg to 1.20 g/Kg (*p*< 0.05), and no marked difference was presented among these three groups.

### 3.4 Analysis of genes expression changes in fish liver

**Figure 2:**
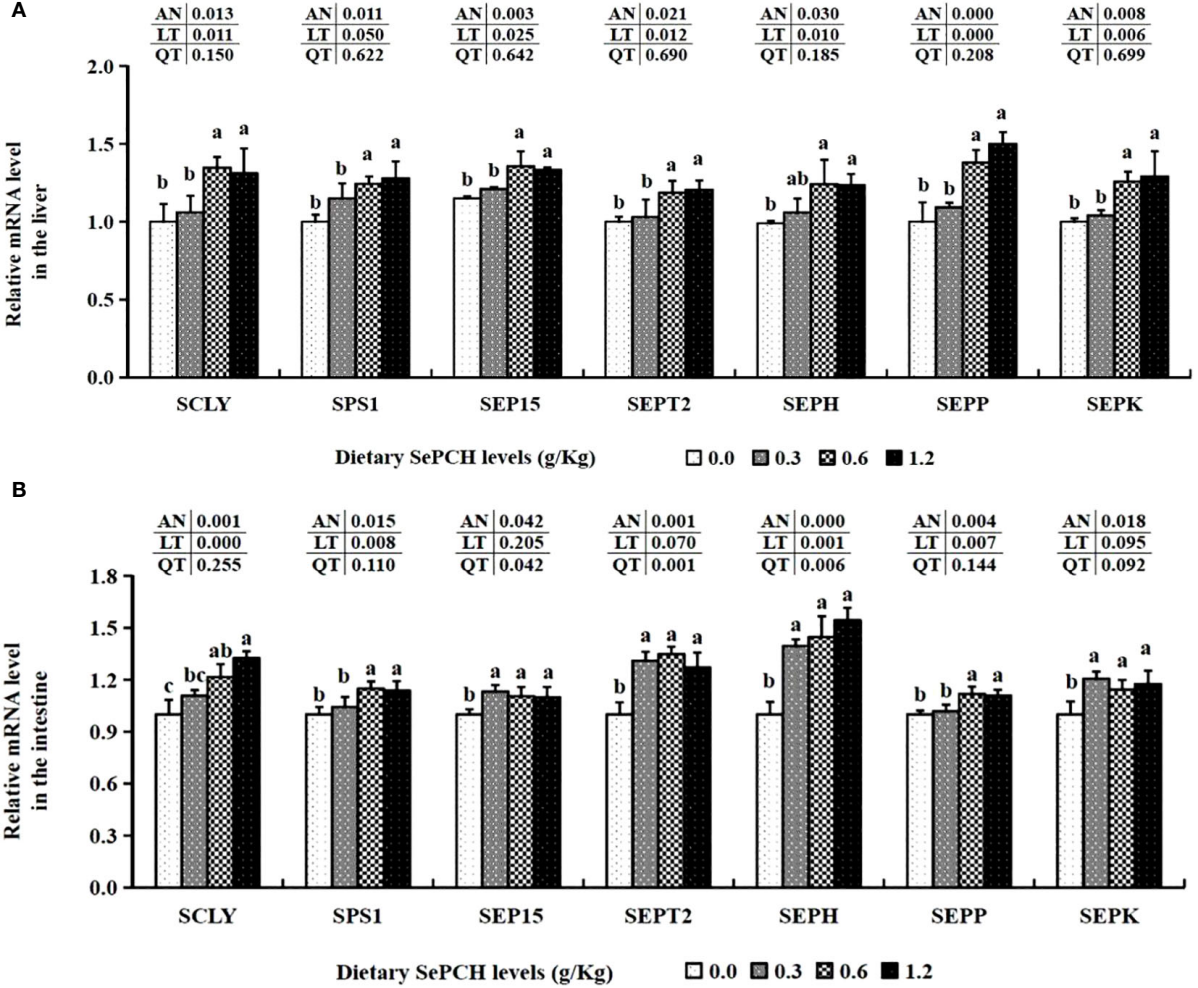
Effect of dietary SePCH on expression levels of selenium metabolism related genes in the liver **(A)** and intestine **(B)** of juvenile largemouth bass.

The mRNA expression levels of these functional genes involved in Se metabolism, such as SCLY and SEP15, were significantly up-regulated in the 0.60 and 1.20 g/Kg SePCH groups compared to the 0.00 and 0.30 g/Kg groups (*p*< 0.05), with no marked difference between 0.60 and 1.20 g/Kg SePCH groups. Moreover, the transcription levels of SEPH in the liver of fish fed with 0.60 and 1.20 g/Kg SePCH were significantly increased in comparation with the controls (*p*< 0.05), and no marked difference was observed compared to the 0.30 g/Kg SePCH groups. With the dosage of SePCH increased, there was a gradual increase in the mRNA transcriptional expression levels of SPS1, SEPT2, SEPP and SEPK. Additionally, the 0.60 and 1.20 g/Kg SePCH markedly increased the mRNA expression levels of SPS1, SEPT2, SEPP and SEPK compared to the 0.00 and 0.30 g/Kg SePCH groups (*p*< 0.05), and there was no significant difference between the 0.60 and 1.20 g/Kg SePCH (**Figure 2A**).

**Figure 3:**
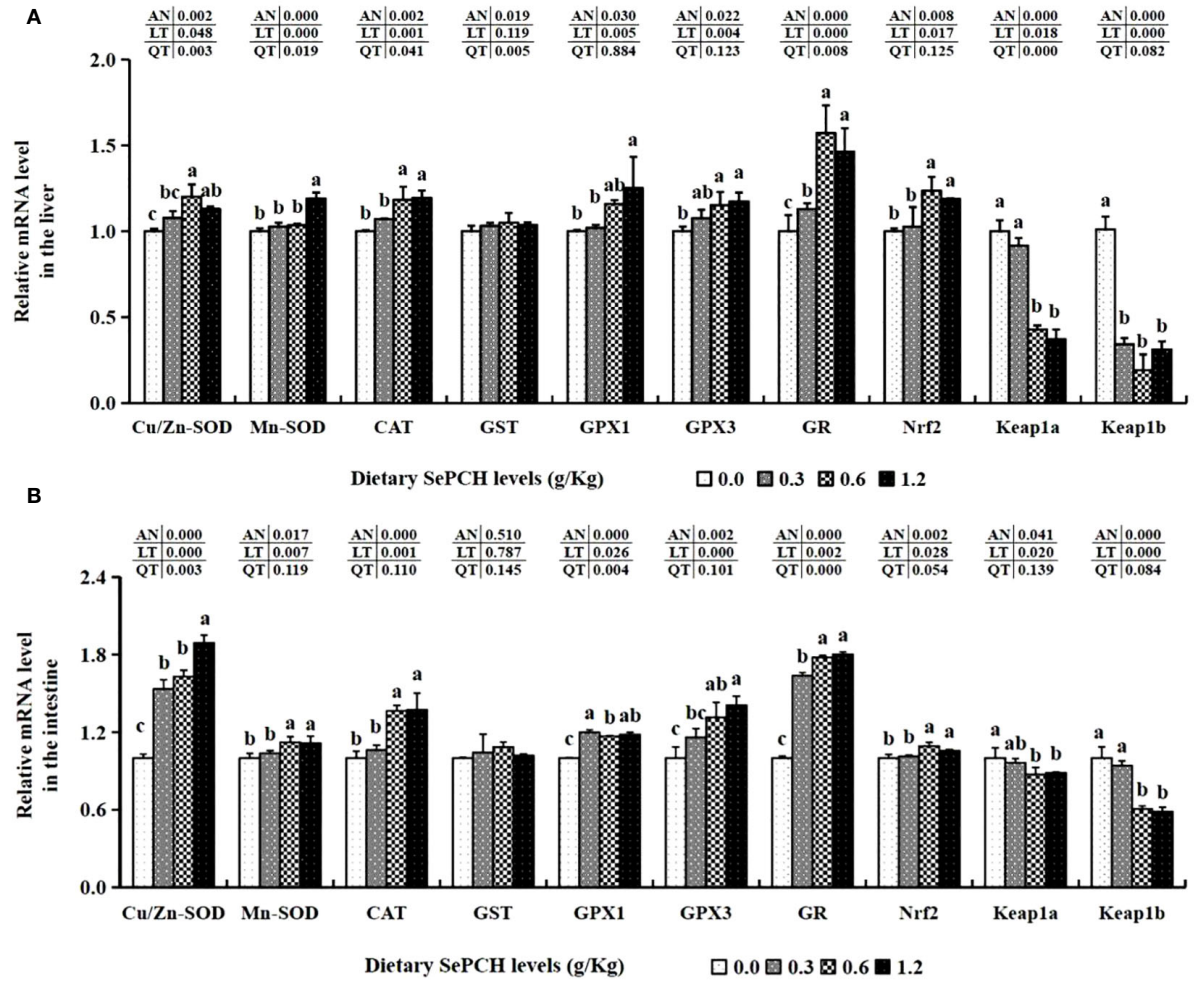
Effect of dietary SePCH on expression levels of antioxidant-related genes in the liver **(A)** and intestine **(B)** of juvenile largemouth bass.

Furthermore, the transcription levels of antioxidant-associated genes, such as Cu/Zn-SOD and GR, in the 0.60 g/Kg SePCH group were notably higher than these in the 0.00 and 0.30 g/Kg SePCH groups (*p*< 0.05), although there was no marked difference compared to the 1.20 g/Kg SePCH groups (**Figure 3A**). The mRNA expression levels of Mn-SOD in the 1.20 g/Kg SePCH groups were markedly higher than that in other three groups (*p*< 0.05), although there were no marked difference among the 0.00, 0.30 and 0.60 SePCH groups. Similarly, the mRNA levels of CAT and GPx1 were notably heightened in the 0.60 and 1.20 g/Kg dietary SePCH groups compared to the 0.00 and 0.30 g/Kg SePCH groups (*p*< 0.05), with no significant difference between the 0.60 and 1.20 g/Kg SePCH groups. The transcription levels of GPx3 in the 0.60 and 1.20 g/Kg SePCH groups were also notably increased compared to the SePCH deficient groups, although there were no marked difference compared to the 0.30 g/Kg SePCH groups. However, no statistical differences on GST mRNA levels were showed among these four SePCH groups (*p* > 0.05). Moreover, the mRNA levels of Nrf2 were notably up-regulated in the 0.60 and 1.20 g/Kg SePCH diets compared to the 0.00 and 0.30 g/Kg SePCH groups (*p*< 0.05). Conversely, the mRNA levels of Keap1a decreased notably in the 0.60 and 1.20 g/Kg SePCH groups compared to the 0.00 and 0.30 g/Kg SePCH groups (*p*< 0.05). Keap1b also showed a gradual decrease from the 0.30 g/Kg to 1.20 g/Kg SePCH groups and reached its lowest levels in the 0.60 g/Kg SePCH groups, which was notably lower than the control groups (**Figure 3A**).

**Figure 4:**
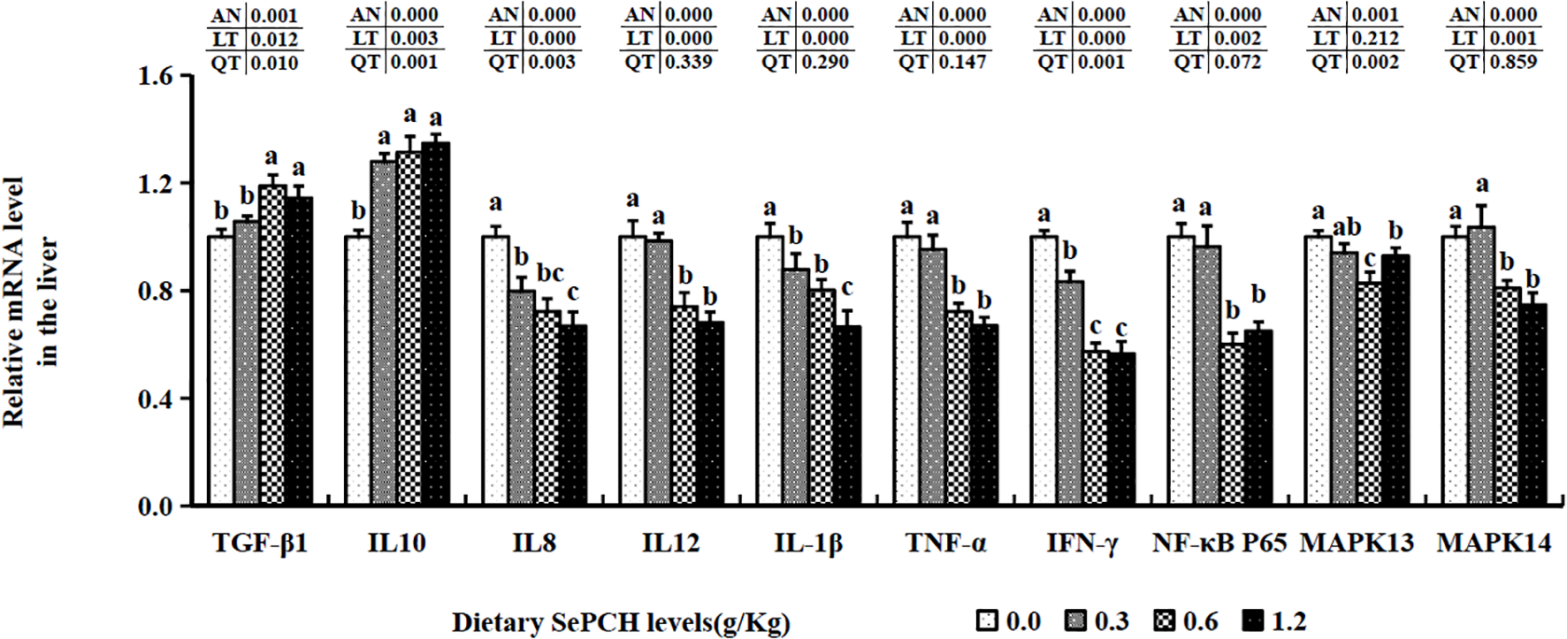
Effect of dietary SePCH on relative gene expression levels of anti-inflammatory, pro-inflammatory cytokines and p38 MAPK/NF-κB signaling pathway in the liver of juvenile largemouth bass.

The transcription levels of TGF-β1 were notably elevated in the 0.60 and 1.20 g/Kg SePCH groups compared to the 0.00 and 0.30 g/Kg SePCH groups (*p*< 0.05), with no marked difference between the 0.60 and 1.20 g/Kg groups (**Figure 4**). Additionally, the mRNA expression levels of IL-10 increased notably in the 0.30 g/Kg to 1.20 g/Kg SePCH groups in comparison with the SePCH deficient groups (*p*< 0.05), and there were no marked differences among these three groups. Nonetheless, 0.60 and 1.20 g/Kg SePCH exhibited a notable reduction in the transcription levels of IL-12 and IFN-γ compared to the 0.00 and 0.30 g/Kg SePCH groups (*p*< 0.05), and no marked difference was presented between the 0.60 and 1.20 g/Kg SePCH groups. While the transcription levels of IL-1β showed a declining trend with increasing doses of SePCH, their levels were significantly reduced only at 1.20 g/Kg SePCH compared with the other groups (*p*< 0.05). Compared to the controls, the transcription levels of IL-8 were significantly reduced in the 0.60 and 1.20 g/Kg SePCH groups, although no marked difference was observed between the 0.60 and 1.20 g/Kg SePCH groups (*p* > 0.05). At the same time, compared to the 0.00 and 0.30 g/Kg SePCH groups, the transcription levels of IFN-γ were notably reduced in the 0.60 and 1.20 g/Kg SePCH groups (*p<* 0.05), and there were no marked difference between the 0.60 and 1.20 g/Kg SePCH groups. Additionally, the transcription levels of NF-κB p65 and MAPK14 were notably reduced in the 0.60 an0 g/Kg SePCH groups compared to the controls (*p*< 0.05), and there were no statistical differences between the 0.60 and 1.20 g/Kg SePCH groups (**Figure 4**). With the increase of SePCH dose, MAPK13 levels showed a decreasing trend and were statistically lower than these in 0.60 g/Kg SePCH groups.

**Figure 5:**
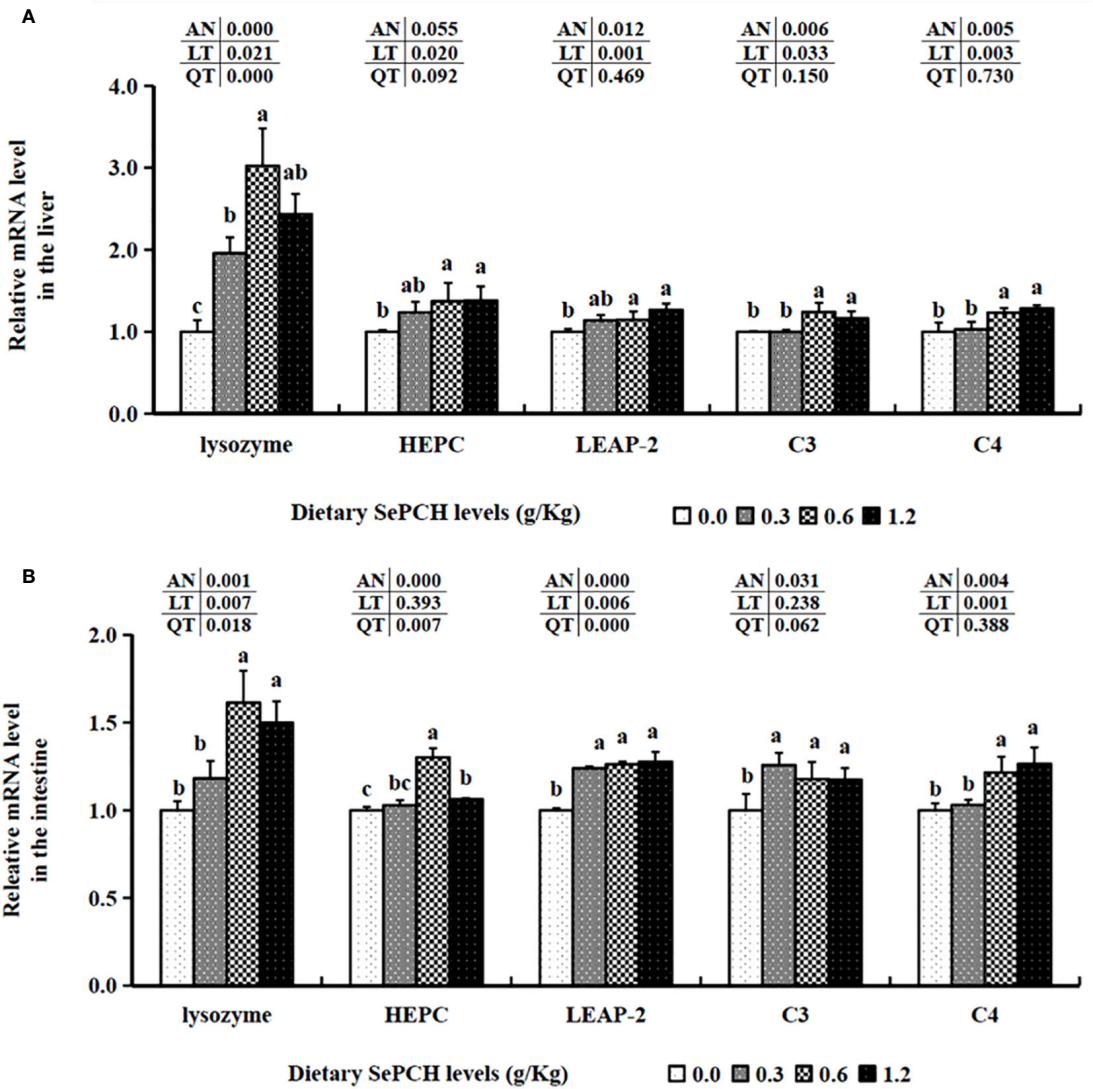
Effect of dietary SePCH on relative gene expression levels of immune-related factors in the liver **(A)** and intestine **(B)** of juvenile largemouth bass.

In terms of immune-related factors, in the 0.00 to 0.60 g/Kg SePCH groups, the expression levels of lysozyme were statistically increased with the increase of SePCH dose (*p*< 0.05) and reached the maximum value in the 0.60 g/Kg SePCH groups, with no marked difference between the 0.60 and 1.20 g/Kg SePCH groups. When the SePCH supplementation was 0.60 and 1.20 g/Kg, the transcription levels of hepcidin (HEPC) and liver-expressed antimicrobial peptide 2 (LEAP-2) were markedly higher than those in the SePCH deficient groups (*p*< 0.05), with no statistical difference between them and the 0.30 g/Kg groups. Furthermore, the transcription levels of C3 and C4 were significantly up-regulated in the 0.60 and 1.20 g/Kg SePCH groups compared to those in the 0.00 and 0.30 g/Kg SePCH groups (*p*< 0.05), and no statistical difference was observed between 0.60 and 1.20 g/Kg SePCH groups (**Figure 5A**).

### 3.5 Genes expression analysis in the intestine

The transcription levels of SCLY in the intestine were significantly elevated and showed a positive correlation with the dietary SePCH levels, with the highest expression observed in the 1.20 g/Kg SePCH groups, significantly surpassing that of the control groups. Furthermore, diets containing 0.60 and 1.20 g/Kg SePCH significantly increased the expression of SPS1 and SEPP in comparison with the controls (*p*< 0.05), with no statistical difference between the 0.60 and 1.20 g/Kg SePCH groups. Simultaneously, diets ranging from 0.30 g/Kg to 1.20 g/Kg SePCH significantly elevated the mRNA expression of SEP15, SEPT2, SEPH and SEPK compared to the SePCH deficient groups (*p*< 0.05), and there were no marked difference among the 0.30, 0.60 and 1.20 g/Kg SePCH groups (**Figure 2B**).

Gene expression assays related to antioxidants in the intestine of our experimental fish revealed a significant increase in Mn-SOD, CAT and GR at dietary SePCH levels of 0.60 and 1.20 g/Kg in comparison with the controls (*p*< 0.05). The transcription levels of GPx3 and Cu/Zn-SOD exhibited a constant increasing trend and reached their peak in the groups fed with 1.20 g/Kg SePCH, significantly exceeding the SePCH deficient groups (*p*< 0.05). Nevertheless, there were not discernible difference in GST mRNA levels in the fish intestine samples in these four experimental groups. The transcription levels of GPx1 exhibited a noteworthy increase in the 0.60 and 1.20 g/Kg SePCH groups (*p*< 0.05) in comparison with the controls, although there were no marked differences between the 0.60 and 1.20 g/Kg SePCH groups. The transcription levels of Nrf2 markedly increased in the groups fed with 0.60 and 1.20 g/Kg SePCH in comparison with the 0.00 and 0.30 g/Kg SePCH groups. The levels of Keap1a and Keap1b significantly decreased in 0.60 and 1.20 g/Kg dietary SePCH in comparison with the SePCH deficient groups (*p*< 0.05), and there were no statistical difference between the 0.60 and 1.20 g/Kg SePCH groups (**Figure 3B**).

**Figure 6:**
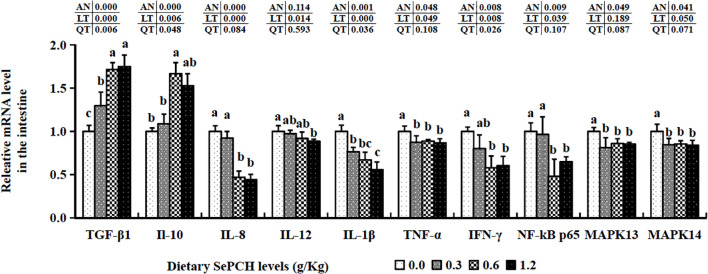
Effect of dietary SePCH on relative gene expression levels of anti-inflammatory, pro-inflammatory cytokines and p38 MAPK/NF-κB signaling pathway in the intestine of juvenile largemouth bass.

The 0.60 and 1.20 g/Kg SePCH groups exhibited a marked increase in the expression levels of TGF-β1 and IL-10 in comparison with the SePCH deficient groups (*p*< 0.05). Importantly, the 0.60 and 1.20 g/Kg SePCH groups showed notable reductions in the transcription levels of IL-8 and IFN-γ in comparison with the SePCH deficient groups (*p*< 0.05). Diets containing 0.30 g/Kg to 1.20 g/Kg SePCH significantly decreased the transcription levels of TNF-α in comparison with the controls (*p*< 0.05), with no marked difference among the 0.30, 0.60 and 1.20 g/Kg SePCH groups. While the transcription levels of IL-12 and IL-1β declined with higher doses of SePCH, the most significant reduction occurred at 1.20 g/Kg SePCH compared to the controls, with no marked difference between the 0.60 and 1.20 g/Kg SePCH groups (**Figure 6**). Additionally, in the 0.60 and 1.20 g/Kg SePCH groups, the transcription levels of NF-κB p65 were considerably reduced in comparison with the SePCH deficient groups (*p*< 0.05). In the 0.30-1.20 g/Kg SePCH groups, the transcription levels of MAPK13 and MAPK14 were all considerably reduced in comparison with the SePCH deficient groups (**Figure 6**).

**Figure 7:**
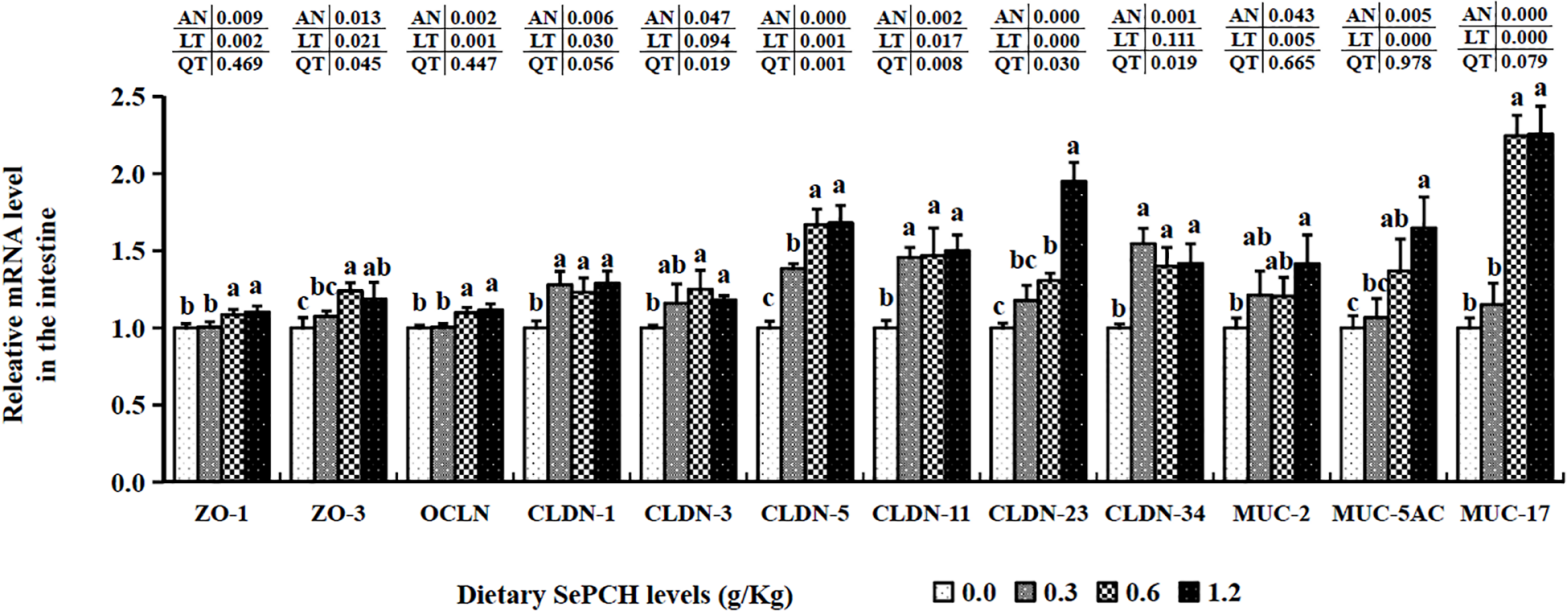
Effects of dietary SePCH on intestinal tight junction-related and intestinal mucins gene expression levels of juvenile largemouth bass.

The transcription levels of HEPC were markedly elevated at the 0.60 g/Kg SePCH groups, significantly higher than in the 0.00, 0.30 and 1.20 g/Kg SePCH groups (**Figure 5B**). Additionally, in the 0.60 and 1.20 g/Kg SePCH groups, the transcription levels of lysozyme and C4 notably increased in comparison with the SePCH deficient groups (*p*< 0.05), with no statistical difference between the 0.60 and 1.20 g/Kg groups (*p* > 0.05). In the 0.30 g/Kg -1.20 g/Kg SePCH groups, the transcription levels of LEAP-2 and C3 were significantly up-regulated in comparison with the control groups (*p*< 0.05). All the transcription levels of CLDN-1, CLDN-11 and CLDN-34 in the intestine showed a discernible increase in the SePCH-treated groups compared to the control groups, although there were no marked differences among these three SePCH treatment groups (*p* > 0.05) (**Figure 7**). Meanwhile, the transcription levels of ZO-1, OCLN, CLDN-3 and CLDN-5 were all significantly increased at 0.60 and 1.20 g/Kg SePCH in comparison with the SePCH deficient groups. In the 0.60 g/Kg SePCH groups, the transcription levels of ZO-3 were markedly raised compared to the 0.00 and 0.30 g/Kg SePCH groups, with no discernible difference in comparison with the 1.20 g/Kg SePCH groups. In addition, mRNA expression levels of CLDN-23 exhibited a consistent increase and were notably higher in the 1.20 g/Kg SePCH groups in comparison with other three groups (*p*< 0.05) (**Figure 7**). While mRNA levels of MUC-2 and MUC-5AC consistently improved with increasing doses of SePCH, there was a significant up-regulation of MUC-2 and MUC-5AC transcription levels only in the 1.20 g/Kg SePCH groups in comparison with the SePCH deficient groups, with no statistical difference between the 0.60 and 1.20 g/Kg groups. The 0.60 and 1.20 g/Kg SePCH groups significantly up-regulated the intestinal MUC-17 genes expressions in comparison with the 0.00 and 0.30 g/Kg SePCH groups (*p*< 0.05) (**Figure 7**).

### 3.6 Histomorphometric analysis of the intestine

**Figure 8:**
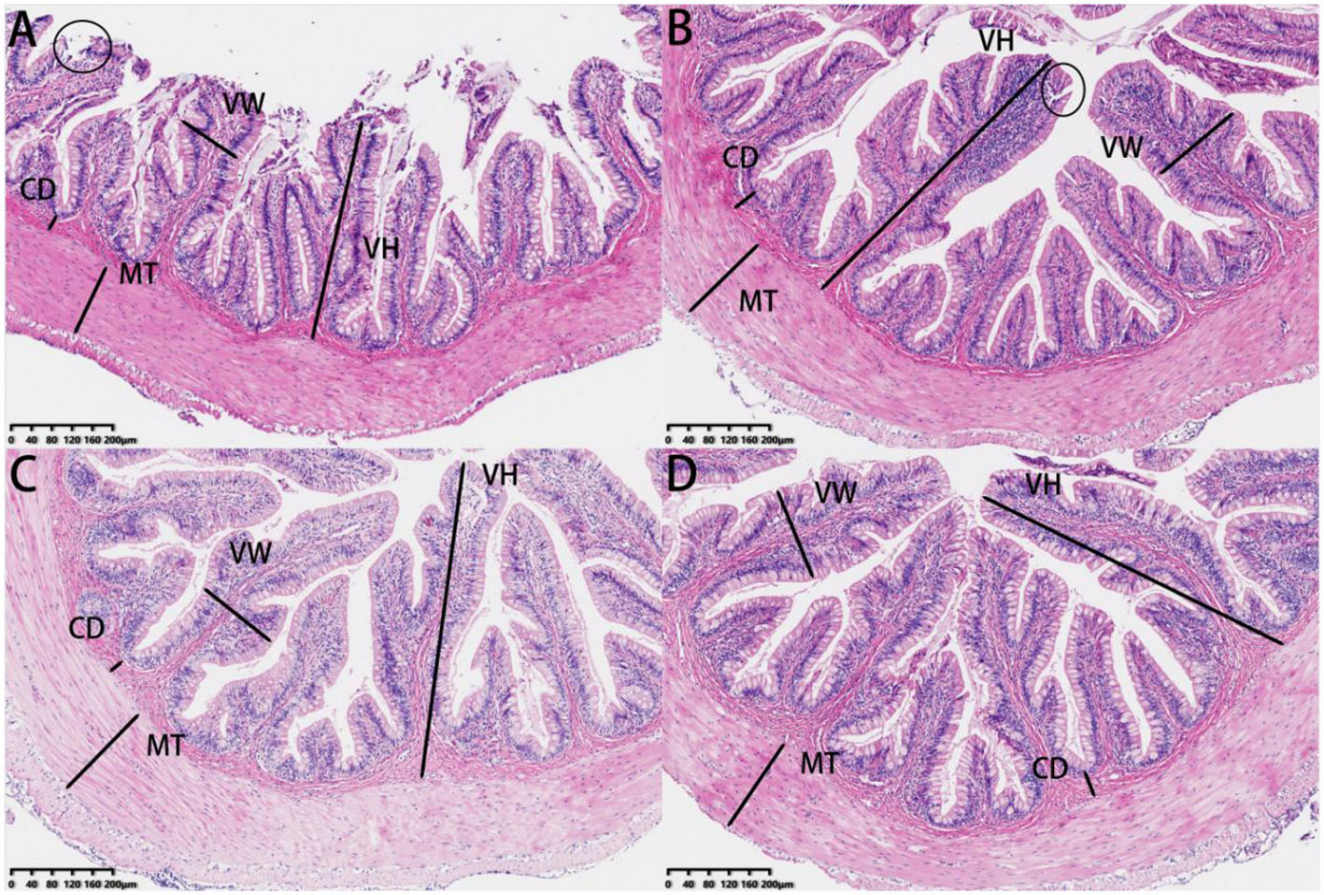
HE staining of the intestine sections of juvenile largemouth bass fed with diets containing 0.00 g/Kg **(A)**, 0.30 g/Kg **(B)**, 0.60 g/Kg **(C)** and 1.20 g/Kg **(D)** SePCH (magnification × 100). VH: villus height, VW, villus width; MT, muscular thickness; CD, crypt depth. Circles represent intestinal villi fall off.

Our investigation of the experimental fish involved the analysis of histological characteristics in intestinal sections (**Figure 8**). The villus height values in the 0.30 g/Kg to 1.20 g/Kg SePCH groups were notably greater than that in the SePCH deficient groups. Meanwhile, the intestinal morphology in the 0.30 g/Kg to 1.20 g/Kg groups showed robust development, characterized by an increased villus height, reaching its peak in the 1.20 g/Kg SePCH groups (*p*< 0.05). In the 0.60 and 1.20 g/Kg SePCH groups, there was a noticeable increase in villus width compared to the 0.00 g/Kg and 0.30 g/Kg groups, and there was no statistical difference between the 0.60 and 1.20 g/Kg SePCH groups (*p* > 0.05). However, there were no marked differences in muscle thickness and crypt depth among these four groups (**Table 7** and **Figure 8**).

**Table 7:** The effects of SePCH on the intestinal morphology of juvenile largemouth bass.

	Supplemental levels of SePCH (g/Kg)						
Parameters	0.00	0.30	0.60	1.20	AN	LT	QT
Villi height (μm)	297.94 ± 4.06 ^b^	596.46 ± 65.71 ^a^	619.70 ± 26.30 ^a^	649.44 ± 11.07 ^a^	0.000	0.004	0.001
Villi width (μm)	94.12 ± 6.12 ^b^	104.73 ± 7.02 ^b^	170.98 ± 19.16 ^a^	179.98 ± 11.23 ^a^	0.000	0.000	0.139
Muscular thickness (μm)	155.76 ± 13.25	154.53 ± 28.96	180.35 ± 26.11	185.89 ± 17.16	0.263	0.065	0.801
Crypt depth (μm)	53.81 ± 10.13	53.80 ± 4.20	47.30 ± 8.93	48.59 ± 12.02	0.751	0.382	0.394

^a–c^ Values in the same row with different letters indicate significant differences (p< 0.05), while that with the same letter or no letter superscripts indicate no significant differences (p > 0.05).

## 4 Discussion

As an essential micronutrient, dietary Se plays crucial roles in promoting growth and maintaining normal physiological functions in different organisms (2, 34). Numerous studies have demonstrated that dietary selenium can influence the growth and development of various aquatic and terrestrial animal species, including largemouth bass (1), European seabass (*Dicentrarchus labrax*) (3), rainbow trout (*Oncorhynchus mykiss*) (7), common carp (*Cyprinus carpio*) (35), grass carp (*Ctenopharyngodon idellus*) (36) and chicken (37). In line with these findings, our results also presented that 0.60 and 1.20 g/Kg SePCH (equivalent to 0.84-1.30 mg/kg) notably heightened WG and DGR, highlighting the role of adequate SePCH (0.60 and 1.20 g/Kg) could enhance the growth performance of largemouth bass (1, 3). As reliable indicators, hematology parameters has been used to evaluate the healthy status and nutritional conditions in many aquatic animals (15, 16). Previous research has shown that higher levels of WBC, MON and LYM can contribute to improved immunity in animals under non-stress conditions (14, 38). In agreement with findings in common carp (39) and Nile tilapia (*Oreochromis niloticus*) (40), our results found that SePCH at concentrations of 0.60 and 1.20 g/Kg increased the counts of WBC, MON and LYM in the blood, suggesting enhanced cellular immune responses in largemouth bass fed with adequate SePCH. Moreover, elevated amounts of RBC, HGB and PLT can enhance oxygen-carrying capacity in animals (41). Similar with results in Nile tilapia (42), African catfish (*Clarias gariepinus*) (43) and European seabass (44), our study found that RBC, HGB, PLT, MCV and MCH levels were also increased by 0.60 and 1.20 g/Kg SePCH, indicating that adequate SePCH can improve oxygen-carrying capacity and cellular immune responses in largemouth bass.

As the primary form of Se found in SePCH (8), selenocysteine can undergo catalysis by SCLY to yield selenide and alanine (45). Subsequently, selenide, in combination with adenosine triphosphate (ATP), can be enzymatically converted into selenophosphate by SPS1 (8, 45). Selenophosphate plays a direct role in the synthesis of various selenoproteins, thus facilitating Se metabolism within organisms (2). Our results shown that 0.60 and 1.20 g/Kg SePCH increased the expression levels of SCLY and SPS1. This observation aligns with previous studies in black carp (*Mylopharyngodon piceus*) (46), Nile tilapia (47) and mice (48). It suggests that elevated SCLY and SPS1 levels induced by sufficient SePCH can enhance the synthesis of selenoproteins and then promote Se metabolism in largemouth bass. SEPP, the sole selenoprotein containing multiple selenocysteine residues, predominantly resides in the plasma, where it acts as a transporter of Se to various tissues (8, 49). Consistent with previous research in triploid rainbow trout (7) and Nile tilapia (47), our study also found higher expression levels of SEPP in fish fed with adequate SePCH, which suggested that dietary SePCH can improve Se transport via SEPP in largemouth bass. SEP15 is localized within the endoplasmic reticulum (ER) lumen, where it can assist in oxidative folding and structural maturation of N-glycosylated proteins for its oxidoreductase activity (50, 51). On the other hand, SEPH can not only play an crucial role in organ development, but also function as a regulator of redox homeostasis for suppressing DNA damage caused by oxidative stress in the nucleus (52, 53). Additionally, as a DNA-binding protein for redox response, SEPH could heighten the expression of functional genes involved in *de novo* GSH synthesis and phase II detoxification for maintaining cellular redox status (54, 55). SEPT is anchored to the membrane of endoplasmic reticulum (ER), where it exerts selenosulfide oxidoreductase activity via its thioredoxin-like domain, serving as a guardian of ER homeostasis (56, 57). Our results demonstrate higher levels of SEP15, SEPT2 and SEPH in fish fed with 0.60 and 1.20 g/Kg SePCH, which aligns with findings in black carp (46), rainbow trout (58) and yellow catfish (*Pelteobagrus fulvidraco*) (59). These findings suggest that adequate SePCH can help maintain ER redox homeostasis by increasing the levels of SEP15, SEPT2 and SEPH in largemouth bass (60). Furthermore, SEPK, an endoplasmic reticulum-membrane protein, plays a crucial role in facilitating effective Ca^2+^ flux during immune cell activation induced by dietary Se (61). In our study, SEPK expression levels were enhanced in the 0.60 and 1.20 g/Kg SePCH groups, consistent with findings in Nile tilapia (47), rainbow trout (58), yellow catfish (59), grass carp (62) and pig (63). These results collectively suggest that adequate SePCH can improve Se metabolism and immune activation by up-regulating the expression of these selenoproteins in largemouth bass.

As is well known, ROS are continually generated during the metabolic processes for all nutrients and can affect changes on the variations of antioxidant enzymes activities in animals and fish (64, 65). Adequate Se can mitigate excessive ROS levels and play vital antioxidant roles through numerous selenoproteins, including Se-dependent glutathione peroxidase (8, 65). In present study, the activities of T-SOD in the liver and intestine were enhanced in fish groups that received sufficient SePCH. These findings are consistent with results reported in largemouth bass (1), European seabass (3), common carp (35) and grass carp (36). Taken together, these results indicate that the higher T-SOD activities induced by adequate SePCH can catalyze the conversion of O_2_^-^ to H_2_O_2_, thus mitigating oxidative stress mediated by O_2_^-^ in largemouth bass. CAT and GSH-Px are the primary antioxidant enzymes responsible for decomposing oxygen radicals by catalyzing the conversion of H_2_O_2_ to H_2_O and O_2_ in animals and fish (8, 9). Similar to previous findings in largemouth bass (1), European seabass (3), Wuchang bream (*Megalobrama amblycephala*) (9) and common carp (35), our results shown that adequate SePCH not only increased the enzyme activities of CAT and GSH-Px but also reduced H_2_O_2_ contents compared to the control groups. This suggests that a sufficient dietary intake of SePCH can promote the breakdown of H_2_O_2_ in largemouth bass. As a dimeric disulfide oxidoreductase, GR can catalyze the reduction of GSH disulfide (GSSG) to GSH for resisting oxidative stress. This process helps maintain the redox environment in human and animal cells (66). Consistent with previous findings in various fish species (46), our study demonstrates higher GR activities and GSH levels in fish fed with adequate SePCH, indicating that sufficient SePCH can enhance cellular redox homeostasis and increase antioxidant capacities by boosting GSH synthesis in the liver and intestine of largemouth bass. Numerous studies have found that the expression of these antioxidant enzymes is primarily regulation by the classical Nrf2/Keap1 signaling transduction pathway (1, 13, 16). Nrf2 can bind Keap1 for maintaining redox homeostasis or antioxidant responses. However, when this balance is disrupted, Nrf2 translocates to the nucleus, where it activates downstream genes related to antioxidant enzymes (13, 67). In this study, we observed up-regulation of Nrf2 and its target molecules, including Cu/Zn-SOD, CAT, GPx1, GPx3 and GR, with a positive correlation to SePCH dosage. However, the expression of Keap1a and Keap1b showed the opposite trend. Similar results were also reported in largemouth bass (1), black carp (46) and chicken (13). Therefore, adequate dietary SePCH can help maintain a better redox status by promoting the transcription levels of antioxidant-related genes through the classical Nrf2/Keap1 signaling transduction pathway in largemouth bass.

ALP and ACP are two essential non-specific phosphohydrolases that can play vital roles in the immune defense through activating immune cells in animals and fish species (5, 17). Similar to findings in grass carp (17), common carp (39) and rainbow trout (68), this investigation observed elevations in ALP and ACP levels in the groups receiving adequate SePCH. Combined with the higher amounts of WBC, MON and LYM in the 0.60 and 1.20 g/Kg SePCH groups, it suggests that adequate SePCH can enhance ALP and ACP activities, thereby stimulating innate immune responses through the activation of these immune cells in largemouth bass. As an antimicrobial enzyme, lysozyme can not only catalyze the hydrolysis of glycosidic bonds in peptidoglycans in the cell walls of gram-positive bacteria, but also acts as an opsonin and play vital roles in facilitating innate immunity by activating the complement system and phagocytes (31). As key constituents in the complement system, C3 and C4 always contribute to both innate and adaptive immunity, making them crucial for host defense against pathogens (16). In this study, the levels of lysozyme, C3 and C4 were all increased in the liver and intestine of fish groups treated with adequate SePCH. These results are consistent with previous findings in black carp (46) and grass carp (17), indicating that adequate SePCH can enhance innate immunity by elevating the levels of lysozyme, C3, and C4 in largemouth bass. As a key element in the humoral immune system, IgM is the primary immunoglobulin present in fish (17). In agreement with results in Wuchang bream (9), common carp (39) and grass carp (17), the contents of IgM were significantly increased in the liver and intestine of fish fed with 0.60 and 1.20 g/Kg SePCH. This suggests that adequate SePCH can enhance humoral immunity via elevating the IgM levels in largemouth bass. HEPC and LEAP-2 are two key antimicrobial proteins expressed in the liver, and they play crucial roles in enhancing immunity by providing the first defense line against many pathogens, including bacteria, fungi, viruses and protozoans (16, 31). Similar to previous results in black carp (46) and largemouth bass (16), our study also found that the transcription levels of HEPC and LEAP-2 were both elevated in the main metabolic and immune tissues of fish fed with adequate dietary SePCH. This indicates the enhanced first-line defense properties mediated by HEPC and LEAP-2 could be enhanced by adequate dietary SePCH in largemouth bass. Taken together, these findings suggest that adequate SePCH can maintain the immunological function homeostasis and provide protective roles for the organism by up-regulating the above defense factors in largemouth bass.

The inflammatory response is a defensive reaction of the immune system to various stimuli such as injury, infection and malnutrition (11). Furthermore, there is a close relationship between the immunological status and the corresponding inflammatory responses, which are primarily controlled by proinflammatory factors (IL-1, IL-8, TNF-α and IFN-γ, etc.) and anti-inflammatory factors (TGF-β1 and IL-10, etc.) (16). In the current study, we observed that adequate SePCH reduced the levels of IL-1β, TNF-α, IL-12, IFN-γ and IL-8 and increased the levels of TGF-β1, along with changes in the transcription levels of these cytokines and IL-10 in the fish liver and intestine. These findings are consistent with previous results in grass carp (24), carp (*C. carpio* var. *specularis*) (69) and European seabass (44). Combined these above findings, it suggest that sufficient dietary SePCH can inhibit the inflammatory response by down-regulating pro-inflammatory genes in the liver and intestine of largemouth bass. It is well-known that the variations in pro-inflammatory and anti-inflammatory cytokines are primarily regulated by the classical p38 MAPK/NF-κB signaling transduction pathway in mammals and animals (4, 11). The p38 MAPK/NF-κB signaling transduction pathway has been illustrated as a crucial pathway involved in various physiological processes, including immune responses, cell survival and inflammation (70). Previous research has shown that inhibiting the p38 MAPK/NF-κB pathways can effectively decrease the transcription and expression levels of pro-inflammatory cytokines, leading to a reduction in inflammatory responses (16, 70). In this study, we observed that the transcription levels of key regulatary elements (MAPK13, MAPK14 and NF-κB p65) were all notably reduced in the liver and intestine of largemouth bass fed with 0.60 and 1.20 g/Kg SePCH in comparison with the SePCH deficient groups, which is similar to earlier studies in grass carp (24), calve (12) and common carp (*C. carpio* L) (71). Collectively, these results indicate that an adequate dietary SePCH can alleviate inflammatory responses by modulating the transcription levels of these pro- and anti-inflammatory genes (IL-1β, TNF-α, IFN-γ, IL-8, IL-12, TGF-β1 and IL-10) through inhibiting the p38 MAPK/NF-κB signaling transduction pathway and thus maintaining immune homeostasis in largemouth bass.

Except as a digestive organ, the intestine also functions as a crucial immune organ, playing vital roles in barrier functions and serving as the first defense line against pathogens’ invasion in fish (31). Previous studies have demonstrated that well-developed intestinal structures are characterized by enhanced villus height and villi width (16). Our research similarly revealed that an adequate supply of SePCH could increase both villus height and villi width in largemouth bass. This suggests that a sufficient dietary intake of SePCH can promote intestinal development and growth (24). As known, the intestinal immune defense relies on the physical barrier integrity formed by the TJ complex, which includes proteins like OCLN, CLDNs and ZOs (22, 31). These proteins serve as crucial indicators for assessing the functional integrity of the intestinal barrier (16, 31). TJ proteins are crucial for maintaining the structural integrity and permeability of the intestinal lining, which is closely linked to the absorption of external nutrients (72). For instance, OCLN functions by penetrating tight junctions, lowering membrane permeability and selectively filtering certain molecules in the animal intestine (73). Meanwhile, as two peripheral membrane functional proteins, ZO-1 and ZO-3 play essential roles for establishing and maintaining tight junctions between endothelial and epithelial cells in the animal intestine (31, 74). Additionally, CLDNs, as transmembrane proteins in cellular tight junctions, are critical for maintaining cell-cell barriers, regulating intercellular communication and preserving cell polarity (22, 75). Consistent with previous findings in largemouth bass (16), grass carp (24), zebrafish (*Danio rerio*) (26), rainbow trout (76) and broiler (77), our results also demonstrate that SePCH at concentrations of 0.60 and 1.20 g/Kg enhances the transcription levels of OCLN, ZO-1, ZO-3, CLDN-1, CLDN-3, CLDN-5, CLDN-11, CLDN-23 and CLDN-34 in the intestine of largemouth bass. This indicates that adequate SePCH can reduce intestinal permeability and bolster the structural integrity of the intestinal barrier (16, 24, 74, 76). Furthermore, MUCs, which are mucosal surface-enriched glycoproteins, play pivotal roles in forming, maintaining and regulating the intestinal barrier and immune responses (78). In our research, we observed an increase in the transcription levels of MUCs (MUC-2, MUC-5AC and MUC-17) in largemouth bass receiving 0.60 and 1.20 g/Kg SePCH. This finding aligns with earlier results in largemouth bass (16), zebrafish (25) and turbot (23), suggesting that adequate SePCH can enhance the mucus barrier, protect the integrity and functionality of the intestinal barrier and maintain health (79). In conclusion, our results indicated that a sufficient dietary intake of SePCH (0.60 and 1.20 g/Kg) can improve intestinal structural integrity and enhance physical barrier function by up-regulating the levels of OCLN, CLDNs and ZOs in largemouth bass.

## 5 Conclusion

In summary, our results found that SePCH at concentrations of 0.60 and 1.20 g/Kg can present several positive effects on largemouth bass. These effects include improvements in growth, hematological indices, Se metabolism and antioxidant capacities through the Nrf2/Keap1 pathway. Additionally, SePCH enhances immunity by increasing the levels of direct defensive factors and mitigates inflammatory responses via the classical p38 MAPK/NF-κB signaling transduction pathway. Moreover, adequate dietary SePCH positively influences intestinal structural integrity and physical barrier function by modulating TJs and MUCs in largemouth bass.

## Data availability statement

The original contributions presented in the study are included in the article/supplementary material. Further inquiries can be directed to the corresponding author.

## Ethics statement

The animal studies were approved by the Experimental Animal Ethics Committee of Huzhou University. The studies were conducted in accordance with the local legislation and institutional requirements. Written informed consent was obtained from the owners for the participation of their animals in this study.

## Author contributions

HZ: Conceptualization, Data curation, Formal analysis, Investigation, Methodology, Software, Validation, Visualization, Writing – original draft, Writing – review & editing. LZ: Data curation, Investigation, Methodology, Validation, Visualization, Writing – original draft, Writing – review & editing. PZ: Formal analysis, Methodology, Software, Validation, Visualization, Writing – original draft, Writing – review & editing, Data curation, Investigation. YX: Data curation, Formal analysis, Investigation, Methodology, Software, Validation, Writing – original draft, Writing – review & editing. XY: Data curation, Formal analysis, Investigation, Methodology, Software, Validation, Writing – original draft. XP: Data curation, Formal analysis, Investigation, Methodology, Software, Writing – original draft. YF: Data curation, Formal analysis, Investigation, Methodology, Software, Writing – original draft. JW: Formal analysis, Investigation, Methodology, Writing – original draft. HB: Resources, Writing – original draft. XS: Supervision, Writing – original draft, Writing – review & editing. JY: Supervision, Writing – review & editing. CW: Conceptualization, Funding acquisition, Methodology, Project administration, Resources, Supervision, Writing – review & editing, Data curation.
